# Lipid Peroxidation: Production, Metabolism, and Signaling Mechanisms of Malondialdehyde and 4-Hydroxy-2-Nonenal

**DOI:** 10.1155/2014/360438

**Published:** 2014-05-08

**Authors:** Antonio Ayala, Mario F. Muñoz, Sandro Argüelles

**Affiliations:** Department of Biochemistry and Molecular Biology, Faculty of Pharmacy, University of Seville, Prof García Gonzales s/n., 41012 Seville, Spain

## Abstract

Lipid peroxidation can be described generally as a process under which oxidants such as free radicals attack lipids containing carbon-carbon double bond(s), especially polyunsaturated fatty acids (PUFAs). Over the last four decades, an extensive body of literature regarding lipid peroxidation has shown its important role in cell biology and human health. Since the early 1970s, the total published research articles on the topic of lipid peroxidation was 98 (1970–1974) and has been increasing at almost 135-fold, by up to 13165 in last 4 years (2010–2013). New discoveries about the involvement in cellular physiology and pathology, as well as the control of lipid peroxidation, continue to emerge every day. Given the enormity of this field, this review focuses on biochemical concepts of lipid peroxidation, production, metabolism, and signaling mechanisms of two main omega-6 fatty acids lipid peroxidation products: malondialdehyde (MDA) and, in particular, 4-hydroxy-2-nonenal (4-HNE), summarizing not only its physiological and protective function as signaling molecule stimulating gene expression and cell survival, but also its cytotoxic role inhibiting gene expression and promoting cell death. Finally, overviews of *in vivo* mammalian model systems used to study the lipid peroxidation process, and common pathological processes linked to MDA and 4-HNE are shown.

## 1. Lipids Overview of Biological Functions****

*Lipids Are Classically Divided into Two Groups: Apolar and Polar.* Triglycerides (apolar), stored in various cells, but especially in adipose (fat) tissue, are usually the main form of energy storage in mammals [1, 2]. Polar lipids are structural components of cell membranes, where they participate in the formation of the permeability barrier of cells and subcellular organelles in the form of a lipid bilayer. The major lipid type defining this bilayer in almost all membranes is glycerol-based phospholipid [3]. The importance of the membrane lipid physical (phase) state is evidenced by the fact that lipids may control the physiological state of a membrane organelle by modifying its biophysical aspects, such as the polarity and permeability. Lipids also have a key role in biology as signaling molecules.

*Lipids as Signaling Molecules*. The main enzymes that generate lipid signaling mediators are lipoxygenase, which mediate hydroperoxyeicosatetraenoic acids (HPETEs), lipoxins, leukotrienes, or hepoxilins biosynthesis after oxidation of arachidonic acid (AA) [4, 5], cyclooxygenase that produces prostaglandins [4], and cytochrome P-450 (CYP) which generates epoxyeicosatrienoic acids, leukotoxins, thromboxane, or prostacyclin [4]. Lipid signaling may occur via activation of a variety of receptors, including G protein-coupled and nuclear receptors. Members of several different lipid categories have been identified as potent intracellular signal transduction molecules. Examples of signaling lipids include (i) two derived from the phosphatidylinositol phosphates, diacylglycerol (DAG) and inositol phosphates (IPs). DAG is a physiological activator of protein kinase C [6, 7] and transcription factor nuclear factor-kB (NF-*κ*B), which promotes cell survival and proliferation. Diacylglycerol also interacts indirectly with other signalling molecules such as small G proteins [8]. IPs are a highly charged family of lipid-derived metabolites, involved in signal transduction that results in activation of Akt, mTOR [9], and calcium-homeostasis [10, 11]; (ii) sphingosine-1-phosphate, a sphingolipid derived from ceramide that is a potent messenger molecule involved in regulating calcium mobilization, migration, adhesion, and proliferation [12–14]; (iii) the prostaglandins, which are one type of fatty-acid derived eicosanoid involved in inflammation [15, 16] and immunity [17]; (iv) phosphatidylserine, a phospholipid that plays an important role in a number of signaling pathways, includes kinases, small GTPases, and fusogenic proteins [18]; (v) the steroid hormones such as estrogen, testosterone, and cortisol, which modulate a host of functions such as reproduction, metabolism, stress response, inflammation, blood pressure, and salt and water balance [19].

## 2. Lipids Damage by Reactive Oxygen Species

One of the consequences of uncontrolled oxidative stress (imbalance between the prooxidant and antioxidant levels in favor of prooxidants) is cells, tissues, and organs injury caused by oxidative damage. It has long been recognized that high levels of free radicals or reactive oxygen species (ROS) can inflict direct damage to lipids. The primary sources of endogenous ROS production are the mitochondria, plasma membrane, endoplasmic reticulum, and peroxisomes [20] through a variety of mechanisms including enzymatic reactions and/or autooxidation of several compounds, such as catecholamines and hydroquinone. Different exogenous stimuli, such as the ionizing radiation, ultraviolet rays, tobacco smoke, pathogen infections, environmental toxins, and exposure to herbicide/insecticides, are sources of* in vivo* ROS production.

**Figure 1:**
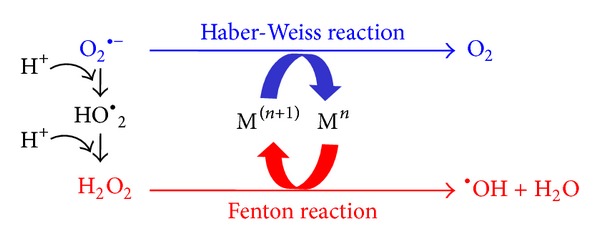
Fenton and Haber-Weiss reaction. Reduced form of transition-metals (M^*n*^) reacts trough the Fenton reaction with hydrogen peroxide (H_2_O_2_), leading to the generation of ^•^OH. Superoxide radical (O_2_^•−^) can also react with oxidized form of transition metals (M^(*n+1*)^) in the Haber-Weiss reaction leading to the production of M^*n*^, which then again affects redox cycling.

The two most prevalent ROS that can affect profoundly the lipids are mainly hydroxyl radical (HO^•^) and hydroperoxyl (HO^•^_2_). The hydroxyl radical (HO^•^) is a small, highly mobile, water-soluble, and chemically most reactive species of activated oxygen. This short-lived molecule can be produced from O_2_ in cell metabolism and under a variety of stress conditions. A cell produces around 50 hydroxyl radicals every second. In a full day, each cell would generate 4 million hydroxyl radicals, which can be neutralized or attack biomolecules [21]. Hydroxyl radicals cause oxidative damage to cells because they unspecifically attack biomolecules [22] located less than a few nanometres from its site of generation and are involved in cellular disorders such as neurodegeneration [23, 24], cardiovascular disease [25], and cancer [26, 27]. It is generally assumed that HO^•^ in biological systems is formed through redox cycling by Fenton reaction, where free iron (Fe^2+^) reacts with hydrogen peroxide (H_2_O_2_) and the Haber-Weiss reaction that results in the production of Fe^2+^ when superoxide reacts with ferric iron (Fe^3+^). In addition to the iron redox cycling described above, also a number of other transition-metal including Cu, Ni, Co, and V can be responsible for HO^•^ formation in living cells (Figure 1).

The hydroperoxyl radical (HO^•^_2_) plays an important role in the chemistry of lipid peroxidation. This protonated form of superoxide yields H_2_O_2_ which can react with redox active metals including iron or copper to further generate HO^•^ through Fenton or Haber-Weiss reactions. The HO^•^_2_ is a much stronger oxidant than superoxide anion-radical and could initiate the chain oxidation of polyunsaturated phospholipids, thus leading to impairment of membrane function [28–30].

### 2.1. Lipid Peroxidation Process

Lipid peroxidation can be described generally as a process under which oxidants such as free radicals or nonradical species attack lipids containing carbon-carbon double bond(s), especially polyunsaturated fatty acids (PUFAs) that involve hydrogen abstraction from a carbon, with oxygen insertion resulting in lipid peroxyl radicals and hydroperoxides as described previously [31]. Glycolipids, phospholipids (PLs), and cholesterol (Ch) are also well-known targets of damaging and potentially lethal peroxidative modification. Lipids also can be oxidized by enzymes like lipoxygenases, cyclooxygenases, and cytochrome P450 (*see above, lipid as signaling molecules*). In response to membrane lipid peroxidation, and according to specific cellular metabolic circumstances and repair capacities, the cells may promote cell survival or induce cell death. Under physiological or low lipid peroxidation rates (subtoxic conditions), the cells stimulate their maintenance and survival through constitutive antioxidants defense systems or signaling pathways activation that upregulate antioxidants proteins resulting in an adaptive stress response. By contrast, under medium or high lipid peroxidation rates (toxic conditions) the extent of oxidative damage overwhelms repair capacity, and the cells induce apoptosis or necrosis programmed cell death; both processes eventually lead to molecular cell damage which may facilitate development of various pathological states and accelerated aging. The impact of lipids oxidation in cell membrane and how these oxidative damages are involved in both physiological processes and major pathological conditions have been analysed in several reviews [32–35].

**Figure 2:**
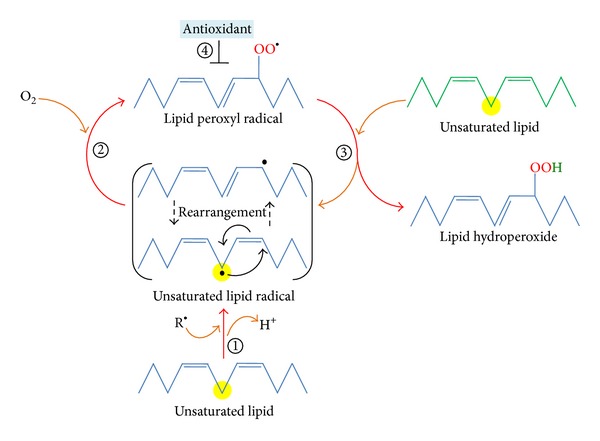
Lipid peroxidation process. In Initiation, prooxidants abstract the allylic hydrogen forming the carbon-centered lipid radical; the carbon radical tends to be stabilized by a molecular rearrangement to form a conjugated diene (step 1). In the propagation phase, lipid radical rapidly reacts with oxygen to form a lipid peroxy radical (step 2) which abstracts a hydrogen from another lipid molecule generating a new lipid radical and lipid hydroperoxide (step 3). In the termination reaction, antioxidants donate a hydrogen atom to the lipid peroxy radical species resulting in the formation of nonradical products (step 4).

The overall process of lipid peroxidation consists of three steps: initiation, propagation, and termination [31, 36, 37]. In the lipid peroxidation initiation step, prooxidants like hydroxyl radical abstract the allylic hydrogen forming the carbon-centered lipid radical (L^•^). In the propagation phase, lipid radical (L^•^) rapidly reacts with oxygen to form a lipid peroxy radical (LOO^•^) which abstracts a hydrogen from another lipid molecule generating a new L^•^ (that continues the chain reaction) and lipid hydroperoxide (LOOH). In the termination reaction, antioxidants like vitamin E donate a hydrogen atom to the LOO^•^ species and form a corresponding vitamin E radical that reacts with another LOO^•^ forming nonradical products (Figure 2). Once lipid peroxidation is initiated, a propagation of chain reactions will take place until termination products are produced. Review with extensive information regarding the chemistry associated with each of these steps is available [31].

### 2.2. Lipid Peroxidation Products

Lipid peroxidation or reaction of oxygen with unsaturated lipids produces a wide variety of oxidation products. The main primary products of lipid peroxidation are lipid hydroperoxides (LOOH). Among the many different aldehydes which can be formed as secondary products during lipid peroxidation, malondialdehyde (MDA), propanal, hexanal, and 4-hydroxynonenal (4-HNE) have been extensively studied by Esterbauer and his colleagues in the 80s [38–49]. MDA appears to be the most mutagenic product of lipid peroxidation, whereas 4-HNE is the most toxic [50].

MDA has been widely used for many years as a convenient biomarker for lipid peroxidation of omega-3 and omega-6 fatty acids because of its facile reaction with thiobarbituric acid (TBA) [48, 51]. The TBA test is predicated upon the reactivity of TBA toward MDA to yield an intensely colored chromogen fluorescent red adduct; this test was first used by food chemists to evaluate autoxidative degradation of fats and oils [52]. However, the thiobarbituric acid reacting substances test (TBARS) is notoriously nonspecific which has led to substantial controversy over its use for quantification of MDA from* in vivo* samples. Several technologies for the determination of free and total MDA, such gas chromatography-mass spectrometry (GC-MS/MS), liquid chromatography-mass spectrometry (LC-MS/MS), and several derivatization-based strategies, have been developed during the last decade [53]. Because MDA is one of the most popular and reliable markers that determine oxidative stress in clinical situations [53], and due to MDA's high reactivity and toxicity underlying the fact that this molecule is very relevant to biomedical research community.

4-HNE was first discovered in 60s [54]. Later, in 80s 4-HNE was reported as a cytotoxic product originating from the peroxidation of liver microsomal lipids [40]. 4-Hydroxyalkenals produced in the course of biomembrane lipids peroxidation, elicited either by free radicals or by chemicals, might exert a genotoxic effect in humans [55]. The 4-hydroxyalkenals are the most significant products because they are produced in relatively large amounts, and they are very reactive aldehydes that act as “second messengers of free radicals.” In particular 4-HNE, which has been subjected to intense scientific scrutiny in 90s [49], is considered as “one of the major toxic products generated from lipid peroxides” [49]. 4-HNE high toxicity can be explained by its rapid reactions with thiols and amino groups [56]. Reactive aldehydes, especially 4-HNE, act both as signaling molecules (*see below 4-HNE as signaling molecule*) and as cytotoxic products of lipid peroxidation causing long-lasting biological consequences, in particular by covalent modification of macromolecules (*see below 4-HNE biomolecular adducts*). 4-HNE is considered as “second toxic messengers of free radicals,” and also as “one of the most physiologically active lipid peroxides,” “one of major generators of oxidative stress,” “a chemotactic aldehydic end-product of lipid peroxidation,” and a “major lipid peroxidation product” [57]. Thus, it is not a surprise that 4-HNE is nowadays considered as major bioactive marker of lipid peroxidation and a signaling molecule involved in regulation of several transcription factors sensible to stress such as nuclear factor erythroid 2-related factor 2 (Nrf2), activating protein-1 (AP-1), NF-*κ*B, and peroxisome-proliferator-activated receptors (PPAR), in cell proliferation and/or differentiation, cell survival, autophagy, senescence, apoptosis, and necrosis (*see below 4-HNE as signaling molecule*).

Characteristics of various lipid peroxidation products as biomarkers have been reviewed on the basis of mechanisms and dynamics of their formation and metabolism and also on the methods of measurement, with an emphasis on the advantages and limitations [58].

### 2.3. Primary Lipid Peroxidation Product-Lipid Hydroperoxides

Hydroperoxides are produced during the propagation phase constituting the major primary product of lipid peroxidation process. The hydroperoxide group may be attached to various lipid structures, for example, free fatty acids, triacylglycerols, phospholipids, and sterols. Lipid hydroperoxide generation, turnover and effector action in biological systems have been reviewed [36]. In contrast to free radical, usually highly reactive and chemically unstable, at moderate reaction conditions, such as low temperature and absence of metal ions, lipid hydroperoxides are relatively more stable products. We found that lipid hydroperoxides in serum could be useful to predict the oxidative stress in tissues [59], and the levels of oxidative stress, including lipid peroxidation, increased throughout the day [60]. Once formed lipid hydroperoxides can be target of different reduction reactions, resulting in peroxidative damage inhibition or peroxidative damage induction.

*Peroxidative Damage Inhibition*. Hydroperoxides may decompose* in vivo* through two-electron reduction, which can inhibit the peroxidative damage. The enzymes mainly responsible for two-electron reduction of hydroperoxides are selenium-dependent glutathione peroxidases (GPx) and selenoprotein P (SeP). GPxs are known to catalyze the reduction of H_2_O_2_ or organic hydroperoxides to water or the corresponding alcohols, respectively, typically using glutathione (GSH) as reductant. Widely distributed in mammalian tissues GPx can be found in the cytosol, nuclei, and mitochondria [61, 62]. The presence of selenocysteine (in the catalytic centre of glutathione peroxidases) as the catalytic moiety was suggested to guarantee a fast reaction with the hydroperoxide and a fast reducibility by GSH [61]. SeP is the major selenoprotein in human plasma that reduced phospholipid hydroperoxide using glutathione or thioredoxin as cosubstrate. It protected plasma proteins against peroxynitrite-induced oxidation and nitration or low-density-lipoproteins (LDL) from peroxidation [62].

*Peroxidative Damage Induction*. Hydroperoxides may also decompose* in vivo* through one-electron reduction and take part in initiation/propagation steps [31, 36, 37], induce new lipid hydroperoxides, and feed the lipid peroxidation process; all these mechanisms can contribute to peroxidative damage induction/expansion. Lipid hydroperoxides can be converted to oxygen radicals intermediates such as lipid peroxyl radical (LOO^•^) and/or alkoxyl (LO^•^) by redox cycling of transition metal (M), resulting in lipid hydroperoxide decomposition and the oxidized or reduced form of theses metal, respectively [63]. The lipid peroxyl and alkoxyl radicals can attack other lipids promoting the propagation of lipid peroxidation

(1) $$\begin{array}{c} \text{LOOH}+{\text{M}}^{n}⟶{\text{LO}}^{•}+{\text{OH}}^{-}+{\text{M}}^{n+1} \end{array}$$

(2) $$\begin{array}{c} \text{LOOH}+{\text{M}}^{n+1}⟶{\text{LOO}}^{•}+{\text{H}}^{+}+{\text{M}}^{n}. \end{array}$$

Lipid hydroperoxides can also react with peroxynitrite (a short-lived oxidant species that is a potent inducer of cell death [64] and is generated in cells or tissues by the reaction of nitric oxide with superoxide radical) or hypochlorous acid (a high reactive species produced enzymatically by myeloperoxidase [65, 66], which utilizes hydrogen peroxide to convert chloride to hypochlorous acid at sites of inflammation) yielding singlet molecular oxygen [67, 68]. Singlet oxygen (molecular oxygen in its first excited singlet state ^1^Δ_*g*_; ^1^O_2_)^1^ can react with amino acid, and proteins resulting in multiple effects including oxidation of side-chains, backbone fragmentation, dimerization/aggregation, unfolding or conformational changes, enzymatic inactivation, and alterations in cellular handling and turnover of proteins [69, 70].

Major substrates for lipid peroxidation are polyunsaturated fatty acids (PUFAs) [31, 36, 37], which are a family of lipids with two or more double bounds, that can be classified in omega-3 (*n*-3) and omega-6 (*n*-6) fatty acids according to the location of the last double bond relative to the terminal methyl end of the molecule. The predominant* n*-6 fatty acid is arachidonic acid (AA), which can be reduced (i) via enzymatic peroxidation to prostaglandins, leukotrienes, thromboxanes, and other cyclooxygenase, lipoxygenase or cytochrome P-450 derived products [4]; or (ii) via nonenzymatic peroxidation to MDA, 4-HNE, isoprostanes, and other lipid peroxidation end-products (more stables and toxic than hydroperoxides) through oxygen radical-dependent oxidative routes [49, 71]. The continued oxidation of fatty acid side-chains and released PUFAs, and the fragmentation of peroxides to produce aldehydes, eventually lead to loss of membrane integrity by alteration of its fluidity which finally triggers inactivation of membrane-bound proteins. Contrary to radicals that attack biomolecules located less than a few nanometres from its site of generation [22], the lipid peroxidation-derived aldehydes can easily diffuse across membranes and can covalently modify any protein in the cytoplasm and nucleus, far from their site of origin [72].

### 2.4. Secondary Lipid Peroxidation Products: MDA

**Figure 3:**
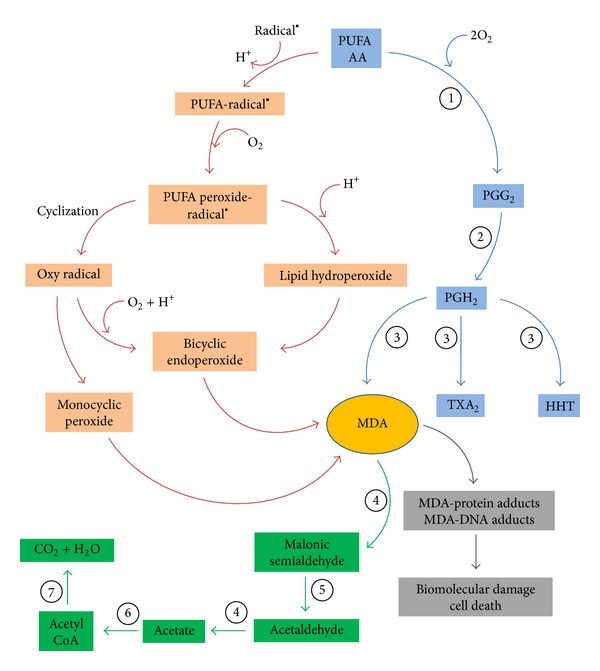
MDA formation and metabolism. MDA can be generated* in vivo* by decomposition of arachidonic acid (AA) and larger PUFAs as a side product by enzymatic processes during the biosynthesis of thromboxane A_2 _(TXA_2_) and 12-l-hydroxy-5,8,10-heptadecatrienoic acid (HHT) (blue pathway), or through nonenzymatic processes by bicyclic endoperoxides produced during lipid peroxidation (red pathway). One formed MDA can be enzymatically metabolized (green pathway). Key enzymes involved in the formation and metabolism of MDA: cyclooxygenases (1), prostacyclin hydroperoxidase (2), thromboxane synthase (3), aldehyde dehydrogenase (4), decarboxylase (5), acetyl CoA synthase (6), and tricarboxylic acid cycle (7).

MDA is an end-product generated by decomposition of arachidonic acid and larger PUFAs [49], through enzymatic or nonenzymatic processes (Figure 3). MDA production by enzymatic processes is well known but its biological functions and its possible dose-dependent dual role have not been studied although MDA is more chemically stable and membrane-permeable than ROS and less toxic than 4-HNE and methylglyoxal (MG) [49]. So far, only few papers have reported that MDA may act as signaling messenger and regulating gene expression: (i) very recent research indicated that MDA acted as a signaling messenger and regulated islet glucose-stimulated insulin secretion (GSIS) mainly through Wnt pathway. The moderately high MDA levels (5 and 10 *μ*M) promoted islet GSIS, elevated ATP/ADP ratio and cytosolic Ca^2+^ level, and affected the gene expression and protein/activity production of the key regulators of GSIS [73]; (ii) in hepatic stellate cells, MDA induced collagen-gene expression by upregulating specificity protein-1 (*Sp1*) gene expression and Sp1 and Sp3 protein levels [74]. Both Sp1 and Sp3 can interact with and recruit a large number of proteins including the transcription initiation complex, histone modifying enzymes, and chromatin remodeling complexes, which strongly suggest that Sp1 and Sp3 are important transcription factors in the remodeling chromatin and the regulation of gene expression [75]. On the other hand, MDA production by nonenzymatic processes remains poorly understood despite their potential therapeutic value, because this MDA is believed to originate under stress conditions and has high capability of reaction with multiple biomolecules such as proteins or DNA that leads to the formation of adducts [76–78], and excessive MDA production have been associated with different pathological states [79–85] (see Table 1). Identifying* in vivo* MDA production and its role in biology is important as indicated by the extensive literature on the compound (over 15 800 articles in the PubMed database using the keyword “malondialdehyde lipid peroxidation” in December 2013).

*MDA Production by Enzymatic Processes*. MDA can be generated* in vivo* as a side product by enzymatic processes during the biosynthesis of thromboxane A_2_ (Figure 3) [86–90]. TXA_2_ is a biologically active metabolite of arachidonic acid formed by the action of the thromboxane A2 synthase, on prostaglandin endoperoxide or prostaglandin H2 (PGH_2_) [4, 91, 92]. PGH_2_ previously is generated by the actions of cyclooxygenases on AA [4, 91, 93].

*MDA Production by Nonenzymatic Processes*. A mixture of lipid hydroperoxides is formed during lipid peroxidation process. The peroxyl radical of the hydroperoxides with a cis-double bond homoallylic to the peroxyl group permits their facile cyclization by intramolecular radical addition to the double bond and the formation of a new radical. The intermediate free radicals formed after cyclization can cyclize again to form bicycle endoperoxides, structurally related to prostaglandins, and undergo cleavage to produce MDA. Through nonenzymatic oxygen radical-dependent reaction, AA is the main precursor of bicyclic endoperoxide, which then undergoes further reactions with or without the participation of other compounds to form MDA (Figure 3) [31, 49, 94, 95]. However, it should be possible that other eicosanoids that can also be generated by nonenzymatic oxygen radical-dependent reaction [96–99] may be precursor of bicyclic endoperoxide and MDA. Recent review has addressed the pathways for the nonenzymatic formation of MDA under specific conditions [100].

*MDA Metabolism*. Once formed MDA can be enzymatically metabolized or can react on cellular and tissular proteins or DNA to form adducts resulting in biomolecular damages. Early studies showed that a probable biochemical route for MDA metabolism involves its oxidation by mitochondrial aldehyde dehydrogenase followed by decarboxylation to produce acetaldehyde, which is oxidized by aldehyde dehydrogenase to acetate and further to CO_2_ and H_2_O (Figure 3) [49, 101, 102]. On the other hand, phosphoglucose isomerase is probably responsible for metabolizing cytoplasmic MDA to methylglyoxal (MG) and further to D-lactate by enzymes of the glyoxalase system by using GSH as a cofactor [103]. A portion of MDA is excreted in the urine as various enaminals (RNH-CH–CH-CHO) such as N-epsilon-(2-propenal)lysine, or N-2-(propenal) serine [49].

#### 2.4.1. MDA Biomolecules Adducts

As a bifunctional electrophile aldehyde, MDA reactivity is pH-dependent, which exists as enolate ion (conjugate bases having a negative charge on oxygen with adjacent C–C double bond) with low reactivity at physiological pH. When pH decreases MDA exists as beta-hydroxyacrolein and its reactivity increases [49]. MDA's high reactivity is mainly based on its electrophilicity making it strongly reactive toward nucleophiles, such as basic amino acid residues (i.e., lysine, histidine, or arginine). Initial reactions between MDA and free amino acids or protein generate Schiff-base adducts [49, 104, 175]. These adducts are also referred to as advanced lipid peroxidation end-products (ALEs). Acetaldehyde (product of MDA metabolism) under oxidative stress and in the presence of MDA further generates malondialdehyde acetaldehyde (MAA) adducts [157, 176]. MAA adducts are shown to be highly immunogenic [177–181]. MDA adducts are biologically important because they can participate in secondary deleterious reactions (e.g., crosslinking) by promoting intramolecular or intermolecular protein/DNA crosslinking that may induce profound alteration in the biochemical properties of biomolecules and accumulate during aging and in chronic diseases [72, 104, 182, 183]. Important proteins that can be modified by MDA adducts are as follows: (i) eElongation factor 2 (eEF2) catalyzes the movement of the ribosome along the mRNA in protein synthesis. MDA adducts with eEF2 could contribute to decline of protein synthesis, secondary to LP increase (*see below—cumene hydroperoxide-induced lipid peroxidation*);* (ii) *factor H (FH) is the main regulator of the alternative pathway in plasma that tightly controls the activation of complement to prevent attack against host cells. MDA adducts with FH can block both the uptake of MDA-modified proteins by macrophages and MDA-induced proinflammatory effects* in vivo* in mice [184]; MDA adducts or MAA adducts can promote binding of complement; (iii) anaphylatoxin C3a (proinflammatory complement components) with oxidatively modified low-density lipoproteins (Ox-LDL) and contributes to inflammatory processes involving activation of the complement system in atherosclerosis [185]; and (iv) protein kinase C (PKC) is known to play a major role in intracellular signal transduction affecting such processes as proliferation, differentiation, migration, inflammation, and cytoskeletal organization. BSA-MAA induces the activation of a specific isoform of PKC, PKC-*α*, in hepatic stellate cells (HSCs) and induces the increased secretion of urokinase-type plasminogen activator, a key component of the plasmin-generating system, thereby contributing to the progression of hepatic fibrosis [186]. A recent review shows a list of up to thirty-three proteins known to be modified by MDA and including enzymatic proteins, carrier proteins, cytoskeletal proteins, and mitochondrial and antioxidant proteins [76].

It has also been proposed that MDA could react physiologically with several nucleosides (deoxy-guanosine and cytidine) to form adducts to deoxyguanosine and deoxyadenosine, and the major product resulting is a pyrimidopurinone called pyrimido[1,2-a]purin-10(3H-)one (M1G or M1dG) [122, 123, 187, 188]. MDA is an important contributor to DNA damage and mutation [122, 124]. The main route for repair of M1dG residues in genomic DNA appears to be the nucleotide excision repair (NER) pathway [188, 189]. In the absence of repair, MDA-DNA adducts may lead to mutations (point and frameshift) [124], strand breaks [122, 190], cell cycle arrest [191], and induction of apoptosis [192]. M1dG is oxidized to 6-oxo-M1dG in rats and that xanthine oxidase (XO) and aldehyde oxidase (AO) are the likely enzymes responsible [193]. This MDA-induced DNA alteration may contribute significantly to cancer and other genetic diseases. Hypermethylated in cancer 1 (HIC1) is a tumor suppressor gene that cooperates with p53 to suppress cancer development. New funding has shown that highest HIC1 methylation levels in tobacco smokers were significantly correlated with oxidative DNA adducts M1dG [125]. Research also suggests that persistent M1dG adducts in mitochondrial DNA hinder the transcription of mitochondrial genes [194]. Dietary intake of certain antioxidants such as vitamins was associated with reduced levels of markers of DNA oxidation (M1dG and 8-oxodG) measured in peripheral white blood cells of healthy subjects, which could contribute to the protective role of vitamins on cancer risk [195].

### 2.5. Secondary Lipid Peroxidation Products: 4-HNE

4-Hydroxynonenal (4-HNE), *α*, *β*-unsaturated electrophilic compounds, is the major type of 4-hydroxyalkenals end-product, generated by decomposition of arachidonic acid and larger PUFAs, through enzymatic or nonenzymatic processes [49]. 4-HNE is an extraordinarily reactive compound containing three functional groups: (i) C=C double bond that can be target to Michael additions to thiol, reduction or epoxidation, (ii) carbonyl group which can yield acetal/thio acetal or can be target to Schiff-base formation, oxidation, or reduction, and (iii) hydroxyl group which can be oxidized to a ketone [56].

4-HNE is the most intensively studied lipid peroxidation end-product, in relation not only to its physiological and protective function as signaling molecule stimulating gene expression, but also to its cytotoxic role inhibiting gene expression and promoting the development and progression of different pathological states. In the last three years, excellent reviews have been published summarizing both signaling and cytotoxic effects of this molecule in biology, for example, overview of mechanisms of 4-HNE formation and most common methods for detecting and analyzing 4-HNE and its protein adducts [196]. Review focuses on membrane proteins affected by lipid peroxidation-derived aldehydes, under physiological and pathological conditions [131]. Jaganjac and Co-workers have described the role of 4-HNE as second messengers of free radicals that act both as signaling molecules and as cytotoxic products of lipid peroxidation involvement in the pathogenesis of diabetes mellitus (DM) [151]. Chapple and Co-workers summarized the production, metabolism and consequences of 4-HNE synthesis within vascular endothelial, smooth muscle cells and targeted signaling within vasculature [142]. Review focuses on the role of 4-HNE and Ox-PLs affecting cell signaling pathways and endothelial barrier dysfunction through modulation of the activities of proteins/enzymes by Michael adducts formation, enhancing the level of protein tyrosine phosphorylation of the target proteins, and by reorganization of cytoskeletal, focal adhesion, and adherens junction proteins [197]. An overview of molecular mechanisms responsible for the overall chemopreventive effects of sulforaphane (SFN), focusing on the role of 4-HNE in these mechanisms, which may also contribute to its selective cytotoxicity to cancer cells [198]. Perluigi and Co-workers summarized the role of lipid peroxidation, particularly of 4-HNE-induced protein modification, in neurodegenerative diseases. In this review, the authors also discuss the hypothesis that altered energy metabolism, reduced antioxidant defense, and mitochondrial dysfunction are characteristic hallmarks of neurodegenerative [170]. Zimniak described the effects of 4-HNE and other endogenous electrophiles on longevity, and its possible molecular mechanisms. The role of electrophiles is discussed, both as destabilizing factors and as signals that induce protective responses [199]. Reed showed the relationship between lipid peroxidation/4-HNE and neurodegenerative diseases. It also demonstrates how findings in current research support the common themes of altered energy metabolism and mitochondrial dysfunction in neurodegenerative disorders [171]. Fritz and Petersen summarized the generation of reactive aldehydes via lipid peroxidation resulting in protein carbonylation, and pathophysiologic factors associated with 4-HNE-protein modification. Additionally, an overview of* in vitro *and* in vivo *model systems used to study the physiologic impact of protein carbonylation, and an update of the methods commonly used in characterizing protein modification by reactive aldehydes [200]. Butterfield and Co-workers showed that several important irreversible protein modifications including protein nitration and 4-HNE modification, both which have been extensively investigated in research on the progression of Alzheimer's disease (AD) [201]. Balogh and Atkins described the cellular effects of 4-HNE, followed by a review of its GST-catalyzed detoxification, with an emphasis on the structural attributes that play an important role in the interactions with alpha-class GSTs. Additionally, a summary of the literature that examines the interplay between GSTs and 4-HNE in model systems relevant to oxidative stress is also discussed to demonstrate the magnitude of importance of GSTs in the overall detoxification scheme [202]. Like MDA, 4-HNE has high capability of reaction with multiple biomolecules such as proteins or DNA that lead to the formation of adducts [49].

**Figure 4:**
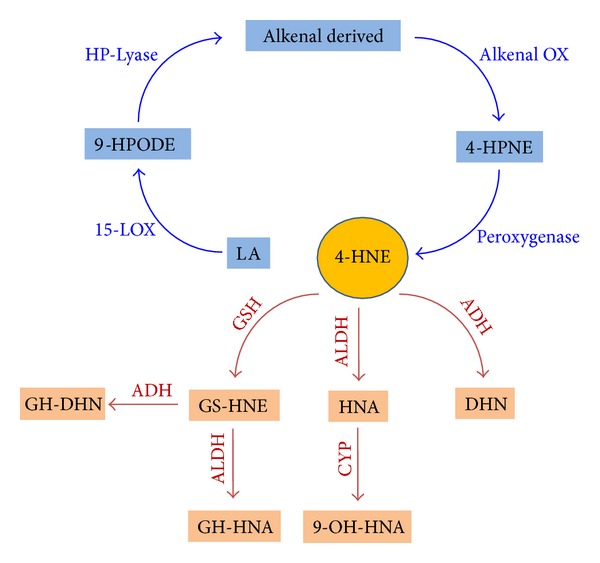
Enzymatic production of 4-HNE and metabolism. In plant enzymatic route to 4-HNE includes lipoxygenase (LOX), -hydroperoxide lyase (HPL), alkenal oxygenase (AKO), and peroxygenases. 4-HNE metabolism may lead to the formation of corresponding alcohol 1,4-dihydroxy-2-nonene (DHN), corresponding acid 4-hydroxy-2-nonenoic acid (HNA), and HNE–glutathione conjugate products. 4-HNE conjugation with glutathione s-transferase (GSH) produce glutathionyl-HNE (GS-HNE) followed by NADH-dependent alcohol dehydrogenase (ADH-)catalysed reduction to glutathionyl-DNH (GS-DNH) and/or aldehyde dehydrogenase (ALDH-)catalysed oxidation to glutathionyl-HNA (GS-HNA). 4-HNE is metabolized by ALDH yielding HNA, which is metabolized by cytochrome P450 (CYP) to form 9-hydroxy-HNA (9-OH-HNA). 4-HNE may be also metabolized by ADH to produce DNH.

*4-HNE Production by Enzymatic Processes*. 4-HNE is a lipid peroxidation end-product of enzymatic transformation of* n*-6 PUFAs (AA, linoleic acid, and other) by 15-lipoxygenases (15-LOX). Two different 15-LOX exist, (i) 15-LOX-1 (reticulocyte type) expressed in reticulocytes, eosinophils, and macrophages; (ii) and 15-LOX-2 (epidermis type) expressed in skin, cornea, prostate, lung, and esophagus [203–205]. Mice do not express 15-LOX and only express the leukocyte-derived 12-LOX. In plant enzymatic route to 4-HNE includes lipoxygenase (*LOX*), -hydroperoxide lyase (*HPL*), alkenal oxygenase (AKO), and peroxygenases (Figure 4) [206]. The main precursors of 4-HNE in human are 13-hydroperoxyoctadecadienoic acid (13-HPODE) produced by the oxidation of linoleic acid by 15-LOX-1 [207] and 15- hydroperoxyeicosatetraenoic acids (15-HPETE) produced by the oxidation of AA by 15-LOX-2 [208]. These compounds are short lived and are catabolised into various families of more stable compounds such as 15-HETEs, lipoxins, and leukotrienes [4]. 15-HPETE is associated with anti-inflammatory and proapoptotic functions (the release of cytochrome* c*, activation of caspase-3 and 8, PARP, and Bid cleavage) and DNA fragmentation [209, 210].

**Figure 5:**
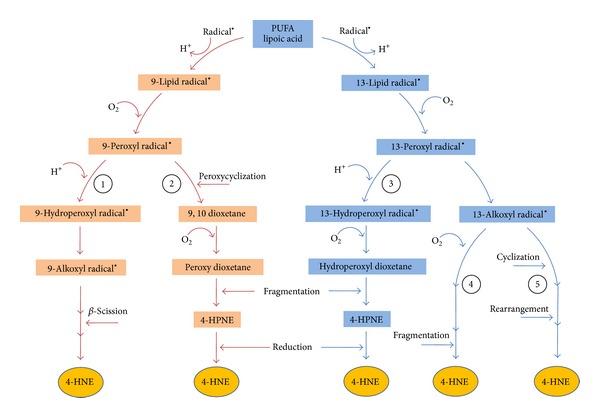
Nonenzymatic 4-HNE production. Initial abstraction of bisallylic hydrogen of lipoic acid (LA) produces fatty radicals. 4-HNE formation starting with 9- and 13-hydroperoxyoctadecadienoate (HPODE) (red and blue pathways, resp.). 4-HNE is generated by beta-scission of a hydroxyalkoxy radical that is produced after cyclization of alkoxy radical in the presence of transition metal ions and two molecules of oxygen; this reaction involves hydrogen abstraction (1). Peroxy radical cyclizes to form a dioxetane which is oxygenated to peroxy-dioxetane that is fragmented and after two hydrogen abstractions produce 4-HNE (2). Hydroperoxyl radical is oxygenated to dioxetane that is further fragmented to produce 4-hydroperoxy-2E-nonenal (4-HPNE), an immediate precursor of 4-HNE (3). Bicyclic endoperoxides react with reduced form of transition metal, such as iron (Fe^2+^) to produce alkoxyl radicals which after reaction with oxygen (O_2_), hydrogen abstraction (H^+^), and fragmentation produce 4-HNE (4). Alkoxyl radical after cyclization, oxygenation, hydrogen abstraction, oxidation of transition metal, hydrolysis, and rearrangement yields 4-HNE (5). With arachidonic acid, 11- and 15- hydroperoxyeicosatetraenoic acids (HPETE) are the precursors to form 4-HNE via the analogous mechanisms.

*4-HNE Production by Nonenzymatic Processes*. 4-HNE can be formed through several nonenzymatic oxygen radical-dependent routes involving the formation of hydroperoxides, alkoxyl radicals, epoxides, and fatty acyl crosslinking reactions. Spickett C [196] recently reviewed the mechanisms of formation of 4-HNE during lipid peroxidation and showed that the main processes leading to 4-HNE are likely beta-cleavage reaction of lipid alkoxy-radicals, which can be* summarized* into five generic mechanisms: (i) reduction of the hydroperoxide to a lipid alkoxy radical by transition metal ions, such as Fe^2+^ followed by b-scission; (ii) protonation of the lipid hydroperoxide yields an acidified lipid hydroperoxide that undergoes Hock rearrangement of a C–C to C–O bond followed by hydrolysis and Hock cleavage; (iii) the lipid peroxyl radical of the hydroperoxides permits their facile cyclization to dioxetane and ending with dioxetane cleavage; (iv) free radical attack to *ω*-6 PUFA on bis-allyl site yielding a free radical intermediate, that further reacts with molecular oxygen to generate hydroperoxide derivatives such as 13-HPODE or 15-HPETE. The abstraction of an allylic hydrogen of their structure produce another radical intermediate that after oxygenation step forms the corresponding dihydroperoxyde derivative (unstable), which after Hock rearrangement and cleavage produces 4-hydroperoxy-2E-nonenal (4S-HPNE), immediate precursor of HNE; and (v) the oxidation products generated after reaction of linoleate-derived hydroperoxy epoxide (13-Hp-Epo-Acid) with Fe^+2^ yields an alkolxyl radical, which undergo to di-epoxy-carbinyl radical and after beta-scission yield different aldehydes compounds including 4-HNE (Figure 5).

**Figure 6:**
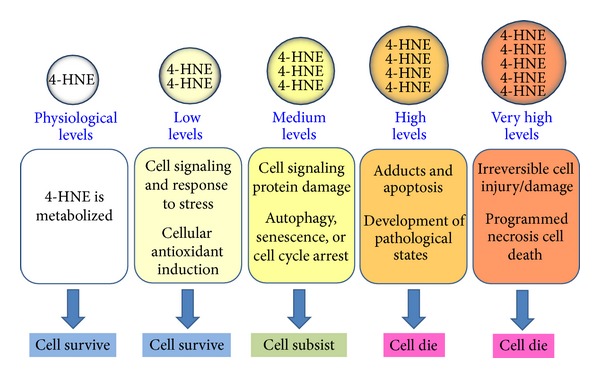
4-HNE promotes cell survival or induces cell death. Depending on cell type, damage/repair capacities and cellular metabolic circumstances 4-HNE can promote cell survival or induce cell death. 4-HNE at physiological levels is enzymatically metabolized and at low levels plays an important role as signaling molecule stimulating gene expression, enhance cellular antioxidant capacity and exert adaptive response; at medium levels organelle and protein damage lead to induction of autophagy, senescence, or cell cycle arrest and at high or very high levels promote adducts formation and apoptosis or necrosis cell death, respectively.

Once formed 4-HNE, and depending of cell type and cellular metabolic circumstances can promote cell survival or death. Cells expressing differentiated functions representative for the* in vivo* situation react more sensitively to 4-HNE than cell lines. The different response with respect to the endpoints of genotoxicity probably depends on the different metabolizing capacities and thus the action of different metabolites of 4-HNE [211]. 4-HNE can be enzymatically metabolized at physiological level and cells can survive; 4-HNE can play an important role as signaling molecule stimulating gene expression (mainly Nrf2) with protective functions that can enhance cellular antioxidant capacity and exert adaptive response when 4-HNE level is low; under this circumstances cells can survive; 4-HNE can promote organelle and protein damage leading to induction of autophagy, senescence, or cell cycle arrest at 4-HNE medium level and cells can subsist; and finally 4-HNE induces apoptosis or necrosis programmed cell death at 4-HNE high or very high level, respectively, and cells die. These processes eventually lead to molecular cell damage which may facilitate development of various pathological states. High levels of 4-HNE can also react with proteins and/or DNA to form adducts resulting in a variety of cytotoxic and genotoxic consequences (Figure 6).

*4-HNE Metabolism*. The main goal of the rapid intracellular metabolism of 4-HNE in mammalian cells is to protect proteins from modification by aldehydic lipid peroxidation products [212]. The biochemical routes of 4-HNE metabolism that lead to the formation of corresponding alcohol 1,4-dihydroxy-2-nonene (DHN), corresponding acid 4-hydroxy-2-nonenoic acid (HNA), and HNE-glutathione conjugate products can be summarized according to stress levels: (i) under physiological or low stress levels the major 4-HNE detoxification step is conjugation with GSH to yield glutathionyl-HNE (GS-HNE) or glutathionyl-lactone (GS-)lactone (cyclic ester 4-HNE- form) followed by NADH-dependent alcohol dehydrogenase (ADH-)catalysed reduction to glutathionyl-DNH (GS-DNH) and/or aldehyde dehydrogenase (ALDH-)catalysed oxidation to glutathionyl-HNA (GS-HNA); (ii) at moderate stress levels, 4-HNE undergoes aldehyde dehydrogenase (ALDH-)catalysed oxidation yielding HNA, that may be further metabolized in mitochondria through beta-oxidation by cytochrome P450 to form 9-hydroxy-HNA; and (iii) at high stress levels, 4-HNE is metabolized by ADH (that belongs to the aldo-keto reductase (AKR) superfamily) to produce DNH [131, 196, 202, 212, 213] (Figure 4). By disrupting the* Gsta*4 gene that encodes the alpha class glutathione s-transferase (GST) isozyme GSTA4-4 in mice showed that GSTA4-4 plays a major role in protecting cells from the toxic effects of oxidant chemicals by attenuating the accumulation of 4-HNE [214]. Overexpression and inhibition of ALDH activity reduce and increase, respectively, the 4-HNE toxicity and 4-HNE-protein adducts levels in cell culture [215, 216].

#### 2.5.1. 4-HNE as Signaling Molecule

At moderate concentration, when the basal level of antioxidant enzymes cannot be sufficient to neutralize 4-HNE, cells can survive due to 4-HNE may regulate several transcription factors sensible to stress such as nuclear factor erythroid 2-related factor 2 (Nrf2), activating protein-1 (AP-1), NF-*κ*B, and peroxisome-proliferator-activated receptors (PPAR). It also activates stress response pathways such as mitogen-activated protein kinases (MAPK), EGFR/Akt pathways, and protein kinase C. Different labs demonstrated the 4-HNE-dependent induction of* Nrf2*, a primary sensor and oxidative stress regulator [217–221]. Also administration of the Nrf2-ARE activators protect from 4-HNE toxicity [222]. Under physiological conditions, Nrf2 is sequestered in the cytoplasm by the repressor protein Keap1, but in response to oxidant stimuli Nrf2 is activated and translocated into the nucleus where mediate the transcription of antioxidant/cytoprotective genes by binding to the antioxidant-response element (ARE) within DNA [223]. The Nrf2-ARE pathway has essential role in different pathological states such as neurodegenerative diseases [223], cancer [224], diabetes [225], and infectious disease [226]. The main genes regulated by 4-HNE- induced Nrf2-ARE pathway are as follows: (i) HO-1, an antioxidant protein that catalyzes the degradation of heme to biliverdin, which is then degraded to bilirubin; both biliverdin and bilirubin have antioxidant properties [227]; 4-HNE can upregulate HO-1 [217, 220, 221, 228–230]; (ii) thioredoxin (Trx) and thioredoxin reductase (TrxR); Trx is a small (13 kDa) antioxidant ubiquitous protein with two redox-active cysteine residues (-Cys-Gly-Pro-Cys-) in its active center; oxidized Trx is reduced back to the active form of Trx by Trx reductase (TrxR) in the presence of NADPH [231]; 4-HNE can upregulate Trx/TrxR [220, 221, 232]; (iii) glutamate cystein ligase (GCL) is a major determinant enzyme in GSH synthesis [233, 234]. 4-HNE can upregulate GCL [235–239].

Involvement of* AP-1* transcription factor in 4-HNE-induced cell signaling has been demonstrated by several studies which showed an AP-1 upregulation by 4-HNE [240–243]. Activation of AP-1 binding may lead to the 4-HNE-induced increase in GSH content [239]. AP-1 is a dimer consisting of basic region-leucine zipper proteins from the Jun and Fos subfamilies. AP-1 transcription factors control cell proliferation, survival, and death. Growth factors, cytokines, cellular stress, and many other stimuli activate AP-1 [244, 245].

*NF-*κ*B* is a dimeric transcription factor that regulates diverse biological processes, including immune responses, inflammation, cell proliferation, and apoptosis. The NF-*κ*B protein complex is retained in an inactive state in the cytoplasm by binding to inhibitory proteins I*κ*Bs family [246]. Various cellular stimuli, such as oxidative stress, I*κ*Bs are phosphorylated, making them susceptible to degradation by the ubiquitin-proteasome system. This results in nuclear translocation of NF-*κ*B complex where it can bind to various promoter areas of its target genes and induce gene transcription of the corresponding genes [246, 247], most of which are implicated in the regulation of inflammation. 4-HNE can activate or inhibit NF-*κ*B depending on the type of cells used. For example, 4-HNE inhibited the activity of NF-*κ*B in hepatocytes [165], cortical neurons [248], ARPE-19 human retinal pigment epithelial cells [249], Kupffer cells [250], human aortic endothelial cells [251], human colorectal carcinoma, and lung carcinoma cell [252]. On the contrary, 4-HNE induced activity of NF-*κ*B in macrophages [253], vascular smooth muscle cells [254], PC12 cells [255], optic nerve head astrocytes [256], human osteoarthritic chondrocytes [257], human fibroblasts [258], and human monocytic lineage cells [259].

*PPARs* comprise three subtypes (PPAR*α*, *β*/*δ*, and *γ*) to form a nuclear receptor superfamily. PPARs act as key transcriptional regulators of lipid metabolism, mitochondrial biogenesis, and antioxidant defense [260, 261]. PPARs interaction/modulation with 4-HNE has been reviewed [262]. 4-HNE increased PPAR-*γ* gene expression and accelerated adiponectin protein degradation in adipocytes [263]; expression of PPAR-*γ* was induced in HL-60 and U937 cells by 4-HNE treatment [264], whereas in the colon cancer cell (CaCo-2) PPAR*γ* protein expression was not induced after 4-HNE treatment [265]; 4-HNE increased PPAR*γ*2 expression in C2C12 cells [266]. PPAR-*β*/*δ* is activated by 4-HNE in 3T3-L1 preadipocytes cells [267]. 4-HNE activates PPAR-*δ* and amplifies insulin secretion in INS-1E *β*-cells [152].

*MAP kinases* family can be activated in response to diverse stimuli such as oxidative stress, lipopolysaccharides, inflammatory cytokines, growth factors, or endoplasmic reticulum (ER) stress and are involved in several cellular responses like cell proliferation and/or differentiation, inflammation, proteasomal-mediated protein degradation, and apoptosis. Members of the major mitogen-activated protein kinase (MAPK) subfamilies are the extracellular signal-regulated kinase (ERK), p38, and Jun N-terminal kinase (JNK) subfamilies. The mechanism by which MAPK signaling cascades are activated by 4-HNE is not well known. For example, activation of different MAPK under various stimuli can affect both apoptotic and prosurvival signaling. In corneal epithelial cells, 4-HNE caused a time-dependent induction of HO-1 mRNA and protein via modification and activation of Erk1/2, JNK and p38 MAP kinases, as well as phosphoinositide-3-kinase (PI3)/Akt. Inhibition of p38 blocked 4-HNE-induced HO-1 expression; inhibition of Erk1/2 and, to a lesser extent, JNK and PI3 K/Akt suppressed 4-HNE-induced HO-1 [268]. 4-HNE also stimulated Erk1/2, JNK, p38, and PI3 kinase in keratinocyte, and the inhibitors of these enzymes suppressed 4-HNE-induced expression of HO-1 [269]. In PC12 cells, 4-HNE treatment induced ERK, JNK, and p38 MAPK activation as well as induced the expression of HO-1. Addition of p38 MAPK specific inhibitor SB203580 attenuated HO-1 upregulation; these results indicate that 4-HNE-induced transient p38 MAPK activation may serve as an upstream negative regulator of ER stress and confer adaptive cytoprotection against 4-HNE-mediated cell injury [228]. In rat liver epithelial RL34 cells, 4-HNE upregulates the cyclooxygenase-2 (COX-2, which plays a key role in conversion of free arachidonic acid to PGs) expression by the stabilization of COX-2 mRNA via activation of the p38 MAPK pathway [270]. In human hepatic stellate cells (hHSC), 4-HNE forms adducts with JNK and this event leads to JNK nuclear translocation and activation as well as to c-jun and AP-1 induction [271]. In human bronchial epithelial cells, 4-HNE downmodulates the protein-tyrosine phosphatase SH2 domain containing phosphatase-1 (SHP-1) which negatively regulates JNK activity [272]. We can also see the protective effects of MAPK activation via GSH induction because the activation of the ERK pathway is involved in GCL (the rate-limiting enzyme in de novo glutathione (GSH) synthesis) regulation in rat cells [273] while the JNK pathways appear to be involved in human HBE-1 cells [274].

In human monocytes, 4-HNE was shown to significantly inhibit p38 and ERK activity, which resulted in inhibition of TNF and interleukin-1beta production in response to LPS. The data suggest that 4-HNE, at nontoxic concentrations, has anti-inflammatory properties [275]. In human osteoarthritic osteoblasts, 4-HNE also showed a significant (approximately 70%) decrease of TNF-*α*-induced IL-6 mRNA expression via the NF-*κ*B signaling pathway. However, only p38 MAPK and JNK1/2 were activated, but not ERK1/2 [276], while 4-HNE also induced COX-2 expression and prostaglandin E2 (PGE2) release [257, 276].

On the other hand, 4-HNE mediated depletion of intracellular thiols, protein tyrosine phosphorylation, MAPK (JNK, ERK, and p38) activation, and modulates integrin resulting in reorganization of cytoskeletal, focal adhesion proteins, and barrier dysfunction in lung microvascular endothelial cells [277]. Results suggest that activation and phosphorylation of MAP kinases (JNK, ERK, and p38) play an important role in 4-HNE mediated toxicity and cell death in mouse embryonic fibroblasts (MEF), and absence of GSTA4–4 potentiates the cytotoxic effects of 4-HNE. The increase of apoptosis in* Gsta*4 null MEF by 4-HNE was associated with the enhanced accumulation of 4-HNE-protein adducts, DNA damage, and the activation of caspases-3, -8, and -9 [214]. 4-HNE upregulates and phosphorylates cytosolic phospholipase A-2 (cPLA-2) in cultured microglial cell line (Ra2) via the ERK and p38 MAPK pathways [278]. cPLA is a proinflammatory enzyme that stimulate AA- release by hydrolyzes glycerophospholipids with AA in the* sn*-2 position.

Matrix metalloproteinases* (MMPs)* constitute a large group of endoproteases that are not only able to cleave all protein components of the extracellular matrix but also to activate or inactivate many other signaling molecules, such as receptors, adhesion molecules, and growth factors [279]. 4-HNE induced MMP-9 production in macrophages [280] and MMP-2 in vascular smooth muscle cells (VSMC) [281] via activation of ERK and p38 MAPK pathways, consequently leading to plaque instability in atherosclerosis. 4-HNE also enhances MMP-2 production in VSMC via mitochondrial ROS-mediated activation of the Akt/NF-kappaB signaling pathways [254]. In osteoarthritic (OA) synovial cells, 4-HNE induced MMP-13 mainly through activation of p38 MAPK [282].

*Akt (a.k.a protein kinase B or PKB)* comprises three closely related isoforms Akt1, Akt2, and Akt3 (or PKB*α*/*β*/*γ* resp.), which play a role in the regulation of cell proliferation, survival, and metabolism. Dysregulation of Akt leads to diseases such as cancer, diabetes, and cardiovascular and neurological diseases [283]. Under conditions of enhanced oxidative stress, a major cellular response is the activation of the Akt pathway that involves the oxidation and subsequent inactivation of PTEN (phosphatase and tensin homolog deleted on chromosome 10), a tumor suppressor and primary regulator of Akt [284]. Recent studies have also demonstrated that activation of PI3 K/Akt signaling by 4-HNE occurs via modification and inhibition of PTEN, a regulatory protein that suppresses Akt2 activity, which is selectively phosphorylated by 4-HNE in both cellular human hepatocellular carcinoma cell line (HepG2) [285] and animal models (ethanol-fed mice) [286]. In HepG2 cells, 4-HNE inhibits H_2_O_2_-mediated activation of the Akt pathway in leading to phosphorylation of Akt1 but not Akt2, decreased cell proliferation, and decreased expression of cyclin D1 [287]. In retinal pigment epithelial (RPE) cells, at lower concentrations 4-HNE triggered phosphorylation of epidermal growth factor receptor (EGFR) and activation of its downstream signaling components ERK1/2 and Akt; this led to protective mechanism against oxidative stress [288]. Akt- induced activity by 4-HNE promotes cell survival through induction of HO-1 mRNA and protein in corneal epithelial cells [268], and in keratinocyte [269]. The inhibitors of Akt suppressed 4-HNE-induced expression of HO-1.

*Protein kinases C (PKCs)* are a family of multifunctional enzymes that play crucial roles in the transduction of many cellular signals such as control of cell proliferation, survival, and transformation by phosphorylating various targets. The PKC family consists of three different groups: conventional (*α*, *β*1, *β*2, and *γ*), novel (*δ*, *ε*, *η*, and *θ*), and atypical (*ζ* and *λ*/*τ*). Conventional and novel PKC isoforms are lipid-sensitive enzymes and calcium-dependent and are usually activated by growth factors through stimulation of phospholipase C (PLC) which hydrolyzes phosphatidylinositol-4,5-bisphosphate (PIP2) to generate inositol triphosphate (IP3) and DAG [6, 289]. Cells can express more than one PKC isoform, and individual PKCs can mediate different biological processes. For example, in human promyelocytic leukemia (HL-60) cells [290–292] and rat neutrophils [293] 4-HNE induced a significant increase of PLC activity, which should result in an increased production of IP3 and DAG, known to stimulate PKC [289]. Phagocytes, such as granulocytes and monocytes/macrophages which engulf microbial intruders and effectively kill and eradicate the foreign bodies, contain a membrane-associated NADPH oxidase that produces superoxide leading to other ROS with microbicidal, tumoricidal, and inflammatory activities [294]. In RAW 264.7 mouse macrophage cells, 4-HNE exhibited a concentration-dependent inhibition of ROS by adduction to PKC, a protein vital in the assembly and activation of NADPH oxidase [295]. In rat hepatocyte PKC- isoforms activity is differentially regulated by concentrations 4-HNE. For example, PKC-*α* activity was decreased in a dose-dependent manner by all concentrations of 4-HNE, while low concentrations of 4-HNE increased PKC *β*I and, to a much greater extent, PKC *β*II activities. By contrast, they were unaffected or even inhibited by higher concentrations of 4-HNE. This PKC-dependent- 4-HNE regulation could be involved in the traffic of secretory glycoproteins [296]. In NT2 neurons, low 4-HNE concentrations (similar to concentrations detected in AD brain tissue) induced a 2–6 fold increase of intracellular amyloid *β*-protein (A*β*) production that was concomitant with selective activation of *β*I and *β*II PKC isoforms [297, 298]. In macrophages, a marked and early upregulation of monocyte chemoattractant protein 1 (MCP-1) release occurs in response to low 4-HNE concentrations, most likely through of the increase in the activity of PKC-*β*I and *β*II classic isoforms, while the activation of PKC-*δ* appeared to be involved in LPS-stimulated cells [299]. Treatment of macrophages with 4-HNE, cell-permeable esters of glutathionyl-4-hydroxynonenal (GS-HNE) and glutathionyl-1,4-dihydroxynonane (GS-DHN) activated NF-*κ*B and PLC/PKC. Aldolase reductase catalyzes the reduction of GS-HNE to GS-DHN. AR inhibition/ablation prevented PLC, PKC, and IKKalpha/beta, and NF-*κ*B activation caused by 4-HNE and GS-HNE, but not by GS-DHN, suggests a novel role for a reduced glutathione-lipid aldehyde conjugate (such as GS-DHN) as an obligatory mediator of ROS-induced cytotoxicity [300].

#### 2.5.2. Effect of 4-HNE on Autophagy

One of the most important processes for maintaining normal metabolic and redox signaling, through degradation of damaged proteins and organelles, is autophagy-lysosomal pathway [301]. 4-HNE can promote protein-adducts leading to protein damage and to induction of autophagy-lysosomal pathway [302], a process that is increased by treatment with an autophagy stimulator, rapamycin. If autophagy is blocked with a PI3 K inhibitor, 3-methyladenine, apoptotic cell death occurs [301, 302]. Several mechanisms by which 4-HNE induces autophagy have been reported. For example, 4-HNE promotes the formation of protein adducts that accumulate in the endoplasmic reticulum (ER) and led to autophagy in rat aortic smooth muscle cells, through selective activation of the PKR-like ER kinase (PERK) pathway accompanied by JNK activation, the upregulation of the HO-1, increased microtubule-associated protein 1 light chain 3 (LC3) formation, and maintenance of cell viability under conditions of excessive 4-HNE-protein adducts accumulation [303]. In differentiated SH-SY5Y neuroblastoma cells, glucose-dependent autophagy serves as a protective mechanism in response to 4-HNE because low 4-HNE-concentrations increased autophagy and induced concentration dependent CASP3/caspase-3 activation and cell death. Additionally inhibition of glucose metabolism by 2-deoxyglucose and glycolysis by koningic acid, a GAPDH, inhibitor, led to autophagy inhibition and increased CASP3 activation and cell death [304]. On the contrary, phagocytosis of 4-HNE- and MDA-modified photoreceptor outer segments (POS) induced a marked reduction of autophagic activity by 40% in retinal pigment epithelium (RPE) cells, which may contribute to RPE cell dysfunction and degeneration. In contrast, unmodified POS had no significant effect on autophagy [305].

#### 2.5.3. Effect of 4-HNE on Senescence

Cellular senescence, defined as arrest during the cell cycle (G0), is involved in the complex process of the biological aging of tissues, organs, and organisms. Senescence is driven by many factors including oxidative stress, the DNA damage/repair response, inflammation, mitogenic signals, and telomere shortening. Telomeres are considered a “biological clock” of the cell and are shortened by each cell division until a critical length is reached and dysfunction ensues. Rapid telomere shortening may indicate a very high cellular activity. DNA-repair pathways are then recruited and cells enter senescence, losing their capacity to proliferate. In addition to cell division, factors causing telomere shortening include DNA damage, inflammation, and oxidative stress [306]. Activation of a DNA damage response including formation of DNA damage foci containing activated H2A.X (*γ*-histone 2A.X) at either uncapped telomeres or persistent DNA strand breaks is the major trigger of cell senescence. *γ*H2AX is a sensitive marker of DNA damage, particularly induction of DNA double-strand breaks [307]. The length of telomeres depends on the telomerase activity and the catalytic subunit of telomerase (hTERT) which is strongly upregulated in most human cancers [308], and the major consequence of the reactivation of telomerase activity is that tumor cells escape from senescence. The expression of c-*myc *(an activator)*, mad-1 *(a repressor) and* sp-1 *(an activator/repressor), which have been shown to activate* hTERT *transcription. The formation of 4-HNE-proteins adducts in general increased as a function of age [309]. Quantitative evaluation showed that the majority of senescent hepatocytes (as measured by *γ*-H2A.X) were also positive for 4-HNE [310, 311]. 4-HNE can induce premature senescence by a direct suppression of telomerase activity affecting the expression of hTERT. In endothelial cells (EC) isolated and cultured from arterial segments of patients with severe coronary artery disease, chronic treatment with an antioxidant (that significantly decreased the levels of lipid peroxidation, that is, 4-HNE expression) N-acetyl-cystein, NAC, significantly delayed cellular senescence via decrease of DNA damage marker (*γ*H2AX), decrease of nuclear p53, and increase in hTERT activity [312]. In three human leukemic cell lines (HL-60, U937, and ML-1) [313] and in colon cancer cells (Caco-2 and HT-29) [314], telomerase activity and hTERT expression were downregulated by 4-HNE, as a consequence of downregulation of c-*myc *mRNA expression and* c-Myc *DNA binding activity as well as upregulation of* mad-1 *mRNA expression and* Mad-1 *DNA binding activity. On the other hand, 4-HNE may induce cellular senescence through activation of critical cell cycle sentinels that mediate this process, such as the tumor suppressor proteins p53 (*see below*), which is well known to play a central role in senescence [315–320]. p53 protects cells of oxidative stress and promotes DNA repair. However, when in the cells the extent of damage overwhelms repair capacities, p53 induces cell death [315–319]. All these data thus confirmed a cell-specific association between senescence and 4-HNE.

#### 2.5.4. Effect of 4-HNE on Cell Cycle and Proliferation

In cell cycle the transition of different phases is driven by several phase-specific cyclin-CDK (cyclin-dependent kinase) complexes which previously have been activated. In response to mitogens, cyclin D is activated and phosphorylate retinoblastoma protein (RB) which leads to activation of E2F proteins and the expression of E2F-responsive genes inducing cells to reenter the cell cycle from quiescence called G0, to G1. Activation of E2F leads to the transcription of cyclin E for transition from G1 to S phase. Subsequent expression of cyclin A leads to transition of S to G2 and cyclin B leads G2 to M phases [321, 322]. The promitotic factor Cdc25 stimulates cell cycle progression through the activation of cyclin A-Cdk1, cyclin B-Cdk1, and cyclin E-Cdk2 for entry into M phase by removing the inhibitory phosphorylation on Cdk1 and Cdk2. On the contrary, the anti-mitotic factor (p21, p27, p57) inhibit cell cycle progression through inhibition of cyclin A–Cdk1, cyclin B–Cdk1, cyclin E–Cdk2 and cyclin D–Cdk4/6 [321–323]. In response to 4-HNE, the expression of key components of cell cycle can be modulated and cells are arrested at G1 or G2. Several studies showed that in general 4-HNE may induce cell cycle arrest in malignant cell and inhibition or decrease of cell proliferation. For example, treatment of HL-60 cells with 4-HNE (1 *μ*M) causes a p53-independent increase of p21 expression, RB dephosphorylation, progressive reduction in the amount of free E2F bound to DNA, and a relative increase in E2F complexes at higher molecular weights with repressive activity decrease of E2F complexes [324], and decrease of cyclin D1, cyclin D2, and cyclin A [325]. In human erythroleukemia cells (K562), 4-HNE treatment increased p53 and p21 expression and decreased expression of cyclin D2. The additional decrease of A- and B-cyclin suggests that the S- and G2-phase were also retarded contributing to the overall slowdown of the cycle [326]. In human breast cancer cells (MCF7) the increase in endogenous levels of 4-HNE caused by treatment with conjugated linoleic acid (CLA) resulted in the inhibition of cell proliferation through a p53-dependent mechanism [327]. In human osteosarcoma cells (HOS), 4-HNE treatment declined gradually the proportion of cells in mitosis, inhibited proliferation and differentiation, and increased apoptosis [328]. In malignant cells like hepatome cells, with a below-normal content of PUFAs and very high expression of aldehyde dehydrogenase-3 (ADH3) which metabolize* 4-HNE* to DNH, the inhibitory effects of 4-HNE on cell proliferation are lower, but the inhibition of ADH3 resulted in an increase in the quantity of aldehyde in the cells and inhibit cell proliferation through the MAPK pathway by reduction of pRaf-1 and pERK1,2 [329, 330]. Moreover, 4-HNE has also antiproliferative/differentiative effect mainly in malignant cell, by affecting the expression of key genes, such as oncogenes (e.g.,* c-myc *and c-*myb*) and cyclins. In three human leukemic cell lines (HL-60, U937, and ML-1) [313] and in colon cancer cells [265, 314], cell proliferation was inhibited by 4-HNE, as a consequence of downregulation of c-*myc* mRNA. 4-HNE mediated inhibition of cell proliferation in the HL-60 cell line by downregulation of Notch1, which is involved in expression of cyclin D1 and c-Myc [331]. In SK-N-BE human neuroblastoma cells, 4-HNE upregulated p53 family gene expression and p53 gene targets p21 and bax, and the consequent reduction in S-phase cells and the increased apoptotic cell proportion; 4-HNE also reduced cyclin D2 expression [332]. In HepG2 cells, 4-HNE decreased both cell survival and proliferation as evidenced by MTT assays and EdU incorporation as well as decreased expression of cyclin D1 and *β*-catenin [287]. In K562 cells [333], HL-60 human leukemic cell line [334], and murine erythroleukemia (MEL) cells [335], 4-HNE inhibited c-myc expression; a oncogene is involved in the regulation of cellular multiplication and transformation (see review of Barrera and co-workers [336]). All these effects increased the proportion of G0/G1 cells, indicating cell cycle arrest at G1 [324, 325, 336, 337]. 4-HNE-induced G2/M cell cycle arrest was via p21 through a mechanism (s) that is independent of p53. The cell cycle arrest leads to apoptotic cell death [338].* Enterococcus faecalis*—infected macrophages produce 4-HNE. This electrophile, when purified, mediated bystander effects in colonic epithelial cells by generating *γ*H2AX foci and inducing G2/M cell cycle arrest. 4-HNE was also associated with mitotic spindle damage, activation of stathmin, cytokinesis failure, and the development of tetraploid [339]. In PC3 prostate cancer cell, 4-HNE induced G2/M cell cycle arrest by decreasing p-Cdc2 (entry into M phase is determined by activation of the Cdc2 protein kinase, which requires Cdc2 dephosphorylation); increased amount of p-H2A.X indicated that 4-HNE induced apoptotic cell death after a G2/M accumulation [340].

In an opposite way, different studies indicated that 4-HNE can promote cell proliferation in normal cells, mainly by upregulation of cyclin or E2F. In cultured primary cortical neurons, 4-HNE increased the protein levels of phospho-p53 and cell cycle-related proteins (cyclin D3, cyclin D1, and CDC25A), caspase-3 activation, PARP cleavage, calpain activation, serine/threonine kinase 3 (Stk3), and sphingosine phosphate lyase 1 (Sgpl1) upregulation. NAC decreased cell death [341]. In smooth muscle cells (SMCs), treatment with 4-HNE enhanced cyclin D1 expression and activation of the ERK signaling pathway, which were stronger in young SMCs compared with aged SMCs [342]. 4-HNE induced vascular smooth muscle cell proliferation [142, 343]. Aldose reductase (AR) efficiently reduces 4-HNE and GS-HNE. Inhibition of AR can arrest cell cycle at S phase. In VSMC cells, the inhibition of AR prevents high glucose (HG-) and/or TNF-alpha-induced VSMC proliferation by accumulating cells at the G1 phase of the cell cycle. Treatment of VSMC with 4-HNE or its glutathione conjugate (glutathionyl (GS-)HNE) or AR-catalyzed product of GS-HNE, GS-1,4-dihydroxynonane resulted in increased E2F-1 expression. Inhibition of AR prevented 4-HNE- or GS-HNE-induced upregulation of E2F-1. Collectively, these results show that AR could regulate HG- and TNF-alpha-induced VSMC proliferation by altering the activation of G1/S-phase proteins such as E2F-1, cdks, and cyclins [344]. In airway smooth muscle cells, 4-HNE is mitogenic by increasing cyclin D1 activity through ERK signaling pathway [345].

The differential effect of 4-HNE on cell proliferation in both malignant and nonmalignant cells may be the consequence of lower aldehyde-metabolizing enzymes, deregulation of antioxidant defenses, and mitochondrial metabolism alteration [132, 346], so that malignant cells are more vulnerable to further oxidative stress induced by exogenous ROS-generating agents or inhibitors of the antioxidant systems [347–349].

#### 2.5.5. 4-HNE-Induced Apoptosis and Necrosis

Apoptosis is essential programmed cell death process for cells, and its dysregulation results in too little cell death which may contribute to carcinogenesis, or too much cell death which may be a component in the pathogenesis of several diseases. The alternative to apoptosis or programmed cell death is necrosis or nonprogrammed cell death, which is considered to be a toxic process where the cell is a passive victim and follows an energy-independent mode of death. Depending on the cell type, DNA damage/repair capacity or cellular metabolic circumstances 4-HNE can activate proliferative signaling for cell division and promote cell survival or “stop” cell division, and after prolonged arrest, cells die from apoptosis. 4-HNE may induce these processes by modulating several transcription factors sensible to stress such as Nrf2, AP-1, NF-*κ*B, and PPAR or by modulating several signaling pathways, including MAPK (p38, Erk, and JNK), protein kinase B, protein kinase C isoforms, cell-cycle regulators, receptor tyrosine kinases, and caspases. Depending on 4-HNE concentrations the cells “end” their lives by apoptosis or necrosis. For example, the cytotoxicity of 4-HNE to HepG2 cells was evaluated by MTT assay. 4-HNE concentrations ranging from 10 to 100 *μ*M gradually decreased cell viability corresponding to an IC_50_ value of 53 ± 2.39 *μ*M. 4-HNE concentrations of 5–40 *μ*M caused apoptotic cell death (measured by flow cytometry, caspase-3 activation, and PARP cleavage). Finally, a significant increase in necrotic cell population, that is, 31.8% and 55.4%, was observed in cells treated with 80 and 100 *μ*M of 4-HNE, respectively [350]. These results show that 4-HNE induces apoptosis at low concentration and necrosis at high concentration.

The two main pathways of apoptosis are extrinsic and intrinsic pathways. The extrinsic signaling pathways that initiate apoptosis involve transmembrane receptor-mediated interactions. This pathway is triggered by the binding of death ligands of the tumor necrosis factor (TNF) family to their appropriate death receptors (DRs) on the cell surface; best-characterized ligands and corresponding death receptors include FasL/FasR and TNF-**α**/TNFR1 [351, 352]. The intrinsic signaling pathways that initiate apoptosis involve a diverse array of non-receptor-mediated stimuli. The proapoptotic member of the Bcl-2 family of proteins, such as Bax, permeabilizes the outer mitochondrial membrane. This allows redistribution of cytochrome c from the mitochondrial intermembrane space into the cytoplasm, where it causes activation of caspase proteases and, subsequently, cell death [352, 353]. Each apoptosis pathway requires specific triggering signals to begin an energy-dependent cascade of molecular events. Each pathway activates its own initiator caspase (8, 9) which in turn will activate the executioner caspase-3 [352]. The execution pathway results in characteristic cytomorphological features including cell shrinkage, chromatin condensation, formation of cytoplasmic blebs and apoptotic bodies, and finally phagocytosis of the apoptotic bodies by adjacent parenchymal cells, neoplastic cells or macrophages [352, 353]. A multitude of mechanisms are employed by p53 to ensure efficient induction of apoptosis in a stage-, tissue-, and stress-signal-specific manner [354]. 4-HNE-mediated activation of p53 may be one of the mechanisms responsible for 4-HNE-induced apoptosis reported in many cell types. For example, in SH-SY5Y cells 4-HNE-induced oxidative stress was associated with increased transcriptional and translational expressions of Bax and p53; these events trigger other processes, ending in cell death [355]. In RPE cells, 4-HNE causes induction, phosphorylation, and nuclear accumulation of p53 which is accompanied with downregulation of MDM2, a negative regulator of the p53 by blocking p53 transcriptional activity directly and mediating in the p53-degradation. Associated proapoptotic genes Bax, p21, and JNK, which are all signaling components p53-mediated pathway of apoptosis, are activated in response to exposure to 4-HNE. The induction of p53 by 4-HNE can be inhibited by the overexpression of either* hGSTA4 *(in RPE cells) or* mGsta4* (in mice) which accelerates disposition of 4-HNE [356]. In CRL25714 cell, 4-HNE induced dose-dependent increase in the expression of p53 in the cytoplasmic and nuclear compartments and increase in the expression of Bax [357]. In human osteoarthritic chondrocytes, 4-HNE treatment led to p53 upregulation, caspase-8, -9, and -3 activation, Bcl-2 downregulation, Bax upregulation, cytochrome c-induced release from mitochondria, poly (ADP-ribose) polymerase cleavage, DNA fragmentation, Fas/CD95 upregulation, Akt inhibition, and energy depletion. All these effects were inhibited by an antioxidant, N-acetyl-cysteine [358].

4-HNE can induce apoptosis through the death receptor Fas (CD95-)mediated extrinsic pathway as well as through the p53-dependent intrinsic pathway. For detailed information of the molecular mechanisms involved in 4-HNE-induced programmed cell death see review [359]. However, these mechanisms can be summarized in the following: (i) 4-HNE is diffusible and can interact with Fas (CD95/Apo1) on plasma membrane and upregulate and activate its expression to mediate the apoptotic signaling through activation of downstream kinases (apoptosis signal-regulating kinase 1 or ASK1 and JNK), which leads to activation of executioner caspase-3 and ending in apoptosis; (ii) 4-HNE interacts with cytoplasmic p53 which causes its induction, phosphorylation, and nuclear translocation. In the nucleus p53 inhibits transcription of antiapoptotic genes (Bcl2) and promotes transcription of proapoptotic genes (Bax) or cell cycle genes (p21) leading to activation of executioner caspase-3 and ending in apoptosis or cell cycle arrest, respectively; (iii) 4-HNE also activates a negative feedback on Fas activation, by a mechanism involving transcription repressor death domain-associated protein (Daxx), a nuclear protein which is associated with DNA-binding transcription factors involved in stress response. 4-HNE interacts with the Daxx, bound to heat shock factor-1 (HSF1), translocates Daxx from nucleus to cytoplasm where it binds to Fas, and inhibits activation of ASK1 to limit apoptosis.

#### 2.5.6. 4-HNE-Biomolecules Adducts

The preference for amino acid modification by 4-HNE is Cys ≫ His > Lys resulting in covalent adducts with the protein nucleophilic side chain [104, 131, 360, 361]. The reaction between primary amines and 4-HNE carbonyl carbon groups yields a reversible Schiff base and the addition of thiol or amino compounds on 4-HNE *β*-carbon atom' (C of double bond) produces the corresponding Michael adduct [49]. 4-HNE-protein adducts can contribute to protein crosslinking and induce a carbonyl stress. Recently it has been shown that a membrane associated protein called regulator of G-protein signaling 4 (RGS4) can be modified by 4-HNE. RGS4, like other RGS proteins, is responsible for temporally regulating G-protein coupled receptor signaling by increasing the intrinsic GTPase activity of G*α* subunit of the heterotrimeric signaling complex. 4-HNE modification of RGS4 at cysteine residues during oxidative stress can disrupt RGS4 activity and alter signaling from stressed cells. Possibly 4-HNE acts as an internal control for aberrant signaling due to excess RGS4 activity in a variety of pathologies where oxidative stress is a strong component [362]. Our lab has reported that 4-HNE can affect protein synthesis rates by forming adduct with eEF2 (*see below—cumene hydroperoxide*-*induced lipid peroxidation*). Large lists of peptides and proteins known to be modified by 4-HNE are given in the reviews [76, 104, 363] and including glutathione, carnosine, enzymatic proteins, carriers proteins, membrane transport proteins, receptor proteins, cytoskeletal proteins, chaperones, mitochondrial upcoupling proteins, transcription and protein synthesis factors, and antioxidant proteins.

It has been reported that 4-HNE also could react with deoxyguanosine to form two pairs of diastereomeres adducts (4-HNE-dG 1,2 and 3,4) that further induced DNA crosslink or DNA-protein conjugates. The mechanism involves a nucleophilic Michael addition of the NH_2_- group of deoxyguanosine to the CC double bond of 4-HNE, which yields 6-(1-hydroxyhexanyl)-8-hydroxy-1,N(2)-propano-2′-deoxyguanosine (HNE-dG), an exocyclic adduct [49, 133, 134]. HNE-dG adducts have been detected in human and animal tissues. They are potentially mutagenic and carcinogenic and can be repaired by the nucleotide excision repair (NER) pathway [364, 365]. In the presence of peroxides a different reaction takes place, and the stable end-product found in the reaction of 4-HNE with DNA bases is etheno-DNA adducts because 4-HNE is converted by the peroxide to the corresponding epoxynonanal, which then reacts to the NH2-group of guanosine followed by cyclization reaction to form 1, N^6^-etheno-2′-eoxyadenosine (*ε*dA), and 3, N^4^-etheno-2′-deoxycytidine (*ε*dC). These *ε*-adducts are eliminated by the base excision repair (BER) pathway [49, 366]. Etheno-DNA adduct levels were found to be significantly elevated in the affected organs of subjects with chronic pancreatitis, ulcerative colitis, and Crohn's disease, which provide promising molecular signatures for risk prediction and potential targets and biomarkers for preventive measures [367, 368]. The 4-HNE-DNA adducts in tissue could serve as marker for the genetic damage produced by endogenous oxidation of omega-6-PUFAs.

## 3. The Use of Mammalian Model in Lipid Peroxidation Research: Compounds Induced Lipid Peroxidation

The use of mammalian model in lipid peroxidation research is ideal for studying the consequences of lipid peroxidation in the context of whole organism and also to analyze their influence on biomarkers to gain more insight into what controls the lipid peroxidation and how lipid peroxidation-related diseases occur. Animal models used to investigate the genetic, physiological, or pathological consequences of lipid peroxidation should try to control the intrinsic and extrinsic influences. Genetic background, diet, environment, and health status can be strictly controlled in many model organisms. Compared with other model organisms, such as worms (*Caenorhabditis elegans*) and flies (*Drosophila melanogaster*), the mammalian model is highly comparable to the human in respect to organ systems, tissues, physiologic systems, and even behavioral traits. Finally, mammalian model in LP can be used as a first step toward possible development of drugs or interventions to control lipid peroxidation process and prevent disease progression in humans. Various mammalian models have been developed to study the lipid peroxidation process.

### 3.1. Cumene Hydroperoxide-Induced Lipid Peroxidation

**Figure 7:**
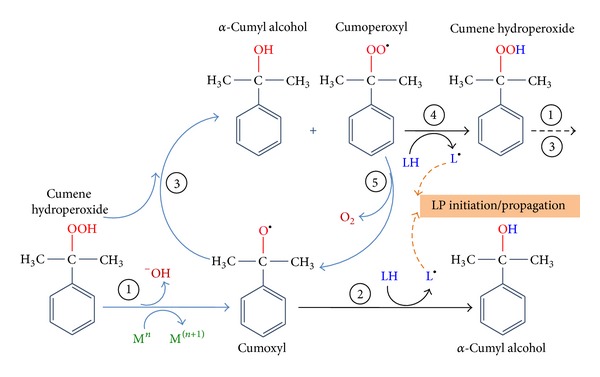
Mechanisms showing how cumene hydroperoxide produces lipophilic cumoxyl and cumoperoxyl radicals. Cumene hydroperoxide in presence of transition metal ions produces cumoxyl radical (step 1), which abstracts a hydrogen (H) from a lipid (PUFA) molecule (LH) generating cumyl alcohol and lipid radical (L^•^) that reacts readily with oxygen promoting the initiation or propagation of lipid peroxidation. (Step 2). Cumoxyl radical can also react with other cumene hydroperoxide molecules to yield cumyl alcohol and cumoperoxyl radical (step 3). Finally, cumoperoxil radical may abstract hydrogen (H) from the closest available lipid to produce a new cumene hydroperoxide and lipid radical (L^•^) which then again affects lipid peroxidation cycling (step 4). Cumoperoxyl radical may also react with oxygen to yield a new cumoxyl radical thus initiating a chain reaction (step 5).

Cumene hydroperoxide (CH) a catalyst used in chemical and pharmaceutical industry [369] is a stable organic oxidizing agent with the peroxy function group, –O–O–, which induces lipid peroxidation. On the existence of transition-metal, CH can be reduced to form an alkoxyl radical, which can attack adjacent fatty acid side-chains to produce lipid radical and cumyl alcohol. The resulting lipid radical reacts with oxygen to form a lipid peroxyl radical. And a lipid peroxyl radical reacts with other fatty acid side-chains to produce a new lipid radical and lipid hydroperoxide and this chain reaction continues. These lipid hydroperoxides may undergo transition-metal mediated one-electron reduction and oxygenation to give lipid peroxyl radicals, which trigger exacerbating rounds of free radical-mediated lipid peroxidation (Figure 7). In our lab we have made extensive use of membrane-soluble CH as a model compound for lipid hydroperoxides (LOOH), which are formed in the process of lipid peroxidation during oxidative stress. CH-induced lipid peroxidation in animals has been important to study the effect of lipid peroxidation on protein synthesis through mechanisms that involve regulation of eElongation Factor 2 (eEF2). It is known that eEF2 plays a key role as a cytoplasmic component of the protein synthesis machinery, where it is a fundamental regulatory protein of the translational elongation step that catalyzes the movement of the ribosome along the mRNA. One particularity of eEF2 is that it is quite sensitive to oxidative stress and is specifically affected by compounds that increase lipid peroxidation, such as cumene hydroperoxide (CH) [370–373]. We have previously reported that cytotoxic end-products of lipid peroxidation 4-HNE and MDA are able to form adducts with eEF2* in vitro* [374] and* in vivo* [309], demonstrating, for the first time, that this alteration of eEF2 could contribute to decline of protein synthesis, secondary to LP increase. The formation of these peroxide-eEF2-adducts is a possible mechanism responsible of suboptimal hormone production from hypothalamic-hypophysis system (HHS) during oxidative stress and aging [375]. The protection of eEF2 alterations by end-products of lipid peroxidation must be specifically carried out by compounds with lipoperoxyl radical-scavenging features such as melatonin. We have reported the ability of melatonin to protect against the changes that occur in the eEF2 under conditions of lipid peroxidation induced by CH, as well as decline of protein synthesis rate caused by lipid peroxidation, demonstrating that melatonin can prevent the decrease of several hormones after exposure to LP [376].* In vitro* studies carried out in our lab also indicated that the antioxidants have different capacities to prevent eEF2 loss caused by CH [377, 378]. In rat hippocampal neurons and in response to lipid peroxidation induced by exposure to CH, eEF2 subcellular localization, abundance, and interaction with p53 were modified [379]. Finally, using CH-induced lipid peroxidation, we found that a unique eEF2 posttranslational modified derivative of histidine (H715) known as diphthamide plays a role in the protection of cells against the degradation of eEF2, and it is important to control the translation of IRES-dependent proteins XIAP and FGF2, two proteins that promote cell survival under conditions of oxidative stress [380]. Other labs have used cumene hydroperoxide as a model compound for lipid hydroperoxides* in vivo* [381–385].

### 3.2. Tert Butyl Hydroperoxide

It is an organic oxidizing agent containing a tertiary butyl group, commonly used in industry as prooxidizing, a bleaching agent, and an initiator of polymerization. Tert butyl hydroperoxide is a strong free radical source and has been utilized to induce lipid peroxidation* in vivo* mammalian model [386–392].

### 3.3. Carbon Tetrachloride (CCl_4_)

It is a toxic, carcinogenic organic compound which is used as a general solvent in industrial degreasing operations. It is also used as pesticides and a chemical intermediate in the production of refrigerants. Carbon tetrachloride has been utilized to induce lipid peroxidation* in vivo* mammalian model [90, 393–398].

### 3.4. Quinolinic Acid (QA)

It is a neuroactive metabolite of the kynurenine pathway. It is normally presented in nanomolar concentrations in human brain and cerebrospinal fluid (CSF) and is often implicated in the pathogenesis of a variety of human neurological diseases [399]. QA has been used to induce lipid peroxidation mediated by hydroxyl radicals* in vivo* mammalian models [400–405].

### 3.5. Transition Metals Ions

They are essential elements which, under certain conditions, can have prooxidant effect. Redox active transition metals have ability to induce and initiate lipid peroxidation through the production of oxygen radicals, mainly hydroxyl radical, via Fenton's/Haber-Weiss reactions [63, 406]. Transition metal, including copper [407–410], chromium [411, 412], cadmium [413–416], nickel [417, 418], vanadium [419–421], manganese [59, 422–424], and iron [59, 407, 425–434] has been utilized to induce lipid peroxidation* in vivo *mammalian model.

## 4. Pathological Processes Linked to MDA and 4-HNE

The accumulation of lipid peroxidation by-product has been extensively studied and implicated in many toxic tissue injuries and in pathological processes. An increasing amount of literature has been published in the field. In particular, the measurement of free MDA and/or 4-HNE levels or its derived protein adducts in biological samples from subjects affected by several diseases has been widely utilized, indirectly implicating MDA and 4-HNE in the pathogenesis of these diseases. Table 1 shows a brief extract of studies presented in the literature in which MDA and 4-HNE have been found to be significantly modified in pathological contexts. The “big” challenge in the field of pathological processes is that it is often difficult to determine whether these lipid peroxidation-derived aldehydes are actually involved in causing the disease or are a consequence to it.

## 5. Conclusions

As conclusion, in this review we summarized the physiological and pathophysiological role of lipid peroxides. When oxidant compounds target lipids, they can initiate the lipid peroxidation process, a chain reaction that produces multiple breakdown molecules, such as MDA and 4-HNE. Among several substrates, proteins and DNA are particularly susceptible to modification caused by these aldehydes. MDA and 4-HNE adducts play a critical role in multiple cellular processes and can participate in secondary deleterious reactions (e.g., crosslinking) by promoting intramolecular or intermolecular protein/DNA crosslinking that may induce profound alteration in the biochemical properties of biomolecules, which may facilitate development of various pathological states. Identification of specific aldehyde-modified molecules has led to the determination of which selective cellular function is altered. For instance, results obtained in our lab suggest that lipid peroxidation affects protein synthesis in all tissues during aging through a mechanism involving the adduct formation of MDA and 4-HNE with elongation factor-2. However, these molecules seem to have a dual behavior, since cell response can tend to enhance survival or promote cell death, depending of their cellular level and the pathway activated by them.

## Figures and Tables

**Table 1:** Common pathological processes linked to MDA and 4-HNE.

Pathological processes	Aldehyde	References
Alzheimer's disease	MDA 4-HNE	[104–113] [81, 108, 114–121]

Cancer	MDA 4-HNE	[109, 122–130] [72, 126–128, 131–136]

Cardiovascular diseases	MDA 4-HNE	[72, 79, 109, 123, 135, 137–141] [72, 104, 109, 131, 135, 138, 139, 142–144]

Diabetes	MDA 4-HNE	[79, 109, 123, 140, 145–150] [131, 135, 142, 143, 151–156]

Liver disease	MDA 4-HNE	[123, 135, 157–164] [135, 160–163, 165–169]

Parkinson's disease	MDA 4-HNE	[81, 108, 114–121] [72, 114, 131, 135, 142, 170–174]

## References

[1] Frühbeck G, Gómez-Ambrosi J, Muruzábal FJ, Burrell MA (2001). The adipocyte: a model for integration of endocrine and metabolic signaling in energy metabolism regulation. *The American Journal of Physiology: Endocrinology and Metabolism*.

[2] Frayn KN (1998). Regulation of fatty acid delivery in vivo. *Advances in Experimental Medicine and Biology*.

[3] Vance E, Vance JE (2002). *Biochemistry: Biochemistry of Lipids, Lipoproteins and Membranes*.

[4] Massey KA, Nicolaou A (2011). Lipidomics of polyunsaturated-fatty-acid-derived oxygenated metabolites. *Biochemical Society Transactions*.

[5] Massey KA, Nicolaou A (2013). Lipidomics of oxidized polyunsaturated fatty acids. *Free Radical Biology and Medicine*.

[6] Jornayvaz FR, Shulman GI (2012). Diacylglycerol activation of protein kinase C*ε* and hepatic insulin resistance. *Cell Metabolism*.

[7] Giorgi C, Agnoletto C, Baldini C (2010). Redox control of protein kinase C: cell-and disease-specific aspects. *Antioxidants and Redox Signaling*.

[8] Yang C, Kazanietz MG (2007). Chimaerins: GAPs that bridge diacylglycerol signalling and the small G-protein Rac. *Biochemical Journal*.

[9] Baumann J, Sevinsky C, Conklin DS (2013). Lipid biology of breast cancer. *Biochimica et Biophysica Acta*.

[10] Fisher SK, Novak JE, Agranoff BW (2002). Inositol and higher inositol phosphates in neural tissues: homeostasis, metabolism and functional significance. *Journal of Neurochemistry*.

[11] Conway SJ, Miller GJ (2007). Biology-enabling inositol phosphates, phosphatidylinositol phosphates and derivatives. *Natural Product Reports*.

[12] Takuwa Y, Okamoto Y, Yoshioka K, Takuwa N (2012). Sphingosine-1-phosphate signaling in physiology and diseases. *BioFactors*.

[13] Mattson MP (2003). *Membrane Lipid Signaling in Aging and Age-Related Disease*.

[14] Hannun YA, Obeid LM (2008). Principles of bioactive lipid signalling: lessons from sphingolipids. *Nature Reviews Molecular Cell Biology*.

[15] Aoki T, Narumiya S (2012). Prostaglandins and chronic inflammation. *Trends in Pharmacological Sciences*.

[16] Tang EHC, Libby P, Vanhoutte PM, Xu A (2012). Anti-inflammation therapy by activation of prostaglandin EP4 receptor in cardiovascular and other inflammatory diseases. *Journal of Cardiovascular Pharmacology*.

[17] Kalinski P (2012). Regulation of immune responses by prostaglandin E_2_. *Journal of Immunology*.

[18] Kay JG, Grinstein S (2013). Phosphatidylserine-mediated cellular signaling. *Advances in Experimental Medicine and Biology*.

[19] Pluchino N, Russo M, Santoro AN, Litta P, Cela V, Genazzani AR (2013). Steroid hormones and BDNF. *Neuroscience*.

[20] Moldovan L, Moldovan NI (2004). Oxygen free radicals and redox biology of organelles. *Histochemistry and Cell Biology*.

[21] Lane N (2002). *Oxygen: The Molecule that Made the World*.

[22] Halliwell B, Gutteridge JMC (1984). Oxygen toxicity, oxygen radicals, transition metals and disease. *Biochemical Journal*.

[23] Venero JL, Revuelta M, Atiki L (2003). Evidence for dopamine-derived hydroxyl radical formation in the nigrostriatal system in response to axotomy. *Free Radical Biology and Medicine*.

[24] Castellani RJ, Honda K, Zhu X (2004). Contribution of redox-active iron and copper to oxidative damage in Alzheimer disease. *Ageing Research Reviews*.

[25] Lipinski B, Pretorius E (2012). Hydroxyl radical-modified fibrinogen as a marker of thrombosis: the role of iron. *Hematology*.

[26] Dizdaroglu M, Jaruga P (2012). Mechanisms of free radical-induced damage to DNA. *Free Radical Research*.

[27] Kanno T, Nakamura K, Ikai H, Kikuchi K, Sasaki K, Niwano Y (2012). Literature review of the role of hydroxyl radicals in chemically-induced mutagenicity and carcinogenicity for the risk assessment of a disinfection system utilizing photolysis of hydrogen peroxide. *Journal of Clinical Biochemistry and Nutrition*.

[28] Bielski BHJ, Arudi RL, Sutherland MW (1983). A study of the reactivity of HO2/O2- with unsaturated fatty acids. *Journal of Biological Chemistry*.

[29] Schneider C, Boeglin WE, Yin H, Porter NA, Brash AR (2008). Intermolecular peroxyl radical reactions during autoxidation of hydroxy and hydroperoxy arachidonic acids generate a novel series of epoxidized products. *Chemical Research in Toxicology*.

[30] Browne RW, Armstrong D (2000). HPLC analysis of lipid-derived polyunsaturated fatty acid peroxidation products in oxidatively modified human plasma. *Clinical Chemistry*.

[31] Yin H, Xu L, Porter NA (2011). Free radical lipid peroxidation: mechanisms and analysis. *Chemical Reviews*.

[32] Volinsky R, Kinnunen PKJ (2013). Oxidized phosphatidylcholines in membrane-level cellular signaling: from biophysics to physiology and molecular pathology. *FEBS Journal*.

[33] Kinnunen PKJ, Kaarniranta K, Mahalka AK (2012). Protein-oxidized phospholipid interactions in cellular signaling for cell death: from biophysics to clinical correlations. *Biochimica et Biophysica Acta*.

[34] Reis A, Spickett CM (2012). Chemistry of phospholipid oxidation. *Biochimica et Biophysica Acta*.

[35] Fruhwirth GO, Loidl A, Hermetter A (2007). Oxidized phospholipids: from molecular properties to disease. *Biochimica et Biophysica Acta: Molecular Basis of Disease*.

[36] Girotti AW (1998). Lipid hydroperoxide generation, turnover, and effector action in biological systems. *Journal of Lipid Research*.

[37] Kanner J, German JB, Kinsella JE (1987). Initiation of lipid peroxidation in biological systems. *Critical Reviews in Food Science and Nutrition*.

[38] Esterbauer H, Cheeseman KH, Dianzani MU (1982). Separation and characterization of the aldehydic products of lipid peroxidation stimulated by ADP-Fe2+ in rat liver microsomes. *Biochemical Journal*.

[39] Poli G, Dianzani MU, Cheeseman KH, Slater TF, Lang J, Esterbauer H (1985). Separation and characterization of the aldehydic products of lipid peroxidation stimulated by carbon tetrachloride or ADP-iron in isolated rat hepatocytes and rat liver microsomal suspensions. *Biochemical Journal*.

[40] Benedetti A, Comporti M, Esterbauer H (1980). Identification of 4-hydroxynonenal as a cytotoxic product originating from the peroxidation of liver microsomal lipids. *Biochimica et Biophysica Acta*.

[41] Cadenas E, Müller A, Brigelius R, Esterbauer H, Sies H (1983). Effects of 4-hydroxynonenal on isolated hepatocytes. Studies on chemiluminescence response, alkane production and glutathione status. *Biochemical Journal*.

[42] Esterbauer H, Lang J, Zadravec S, Slater TF (1984). Detection of malonaldehyde by high-performance liquid chromatography. *Methods in Enzymology*.

[43] Winkler P, Lindner W, Esterbauer H, Schauenstein E, Schaur RJ, Khoschsorur GA (1984). Detection of 4-hydroxynonenal as a product of lipid peroxidation in native Ehrlich ascites tumor cells. *Biochimica et Biophysica Acta: Lipids and Lipid Metabolism*.

[44] Esterbauer H, Benedetti A, Lang J, Fulceri R, Fauler G, Comporti M (1986). Studies on the mechanism of formation of 4-hydroxynonenal during microsomal lipid peroxidation. *Biochimica et Biophysica Acta: Lipids and Lipid Metabolism*.

[45] Hurst JS, Slater TF, Lang J (1987). Effects of the lipid peroxidation product 4-hydroxynonenal on the aggregation of human platelets. *Chemico-Biological Interactions*.

[46] Cheeseman KH, Beavis A, Esterbauer H (1988). Hydroxyl-radical-induced iron-catalysed degradation of 2-deoxyribose. Quantitative determination of malondialdehyde. *Biochemical Journal*.

[47] Esterbauer H, Zolliner H (1989). Methods for determination of aldehydic lipid peroxidation products. *Free Radical Biology and Medicine*.

[48] Esterbauer H, Cheeseman KH (1990). Determination of aldehydic lipid peroxidation products: malonaldehyde and 4-hydroxynonenal. *Methods in Enzymology*.

[49] Esterbauer H, Schaur RJ, Zollner H (1991). Chemistry and Biochemistry of 4-hydroxynonenal, malonaldehyde and related aldehydes. *Free Radical Biology and Medicine*.

[50] Esterbauer H, Eckl P, Ortner A (1990). Possible mutagens derived from lipids and lipid precursors. *Mutation Research*.

[51] Pryor WA (1989). On the detection of lipid hydroperoxides in biological samples. *Free Radical Biology and Medicine*.

[52] Sinnhuber RO, Yu TC, Yu TC (1958). Characterization of the red pigment formed in the 2-thiobarbituric acid determination of oxidative rancidity. *Journal of Food Science*.

[53] Giera M, Lingeman H, Niessen WMA (2012). Recent advancements in the LC- and GC-based analysis of malondialdehyde (MDA): a brief overview. *Chromatographia*.

[54] Schauenstein E (1967). Autoxidation of polyunsaturated esters in water: chemical structure and biological activity of the products. *Journal of Lipid Research*.

[55] Brambilla G, Sciabà L, Faggin P (1986). Cytotoxicity, DNA fragmentation and sister-chromatid exchange in Chinese hamster ovary cells exposed to the lipid peroxidation product 4-hydroxynonenal and homologous aldehydes. *Mutation Research*.

[56] Schaur RJ (2003). Basic aspects of the biochemical reactivity of 4-hydroxynonenal. *Molecular Aspects of Medicine*.

[57] Zarkovic N (2003). 4-Hydroxynonenal as a bioactive marker of pathophysiological processes. *Molecular Aspects of Medicine*.

[58] Niki E (2014). Biomarkers of lipid peroxidation in clinical material. *Biochimica et Biophysica Acta*.

[59] Argüelles S, García S, Maldonado M, Machado A, Ayala A (2004). Do the serum oxidative stress biomarkers provide a reasonable index of the general oxidative stress status?. *Biochimica et Biophysica Acta: General Subjects*.

[60] Argüelles S, Gómez A, Machado A, Ayala A (2007). A preliminary analysis of within-subject variation in human serum oxidative stress parameters as a function of time. *Rejuvenation Research*.

[61] Brigelius-Flohé R, Maiorino M (2013). Glutathione peroxidases. *Biochimica et Biophysica Acta*.

[62] Steinbrenner H, Sies H (2009). Protection against reactive oxygen species by selenoproteins. *Biochimica et Biophysica Acta: General Subjects*.

[63] Valko M, Morris H, Cronin MTD (2005). Metals, toxicity and oxidative stress. *Current Medicinal Chemistry*.

[64] Szabó C, Ischiropoulos H, Radi R (2007). Peroxynitrite: biochemistry, pathophysiology and development of therapeutics. *Nature Reviews Drug Discovery*.

[65] Winterbourn CC (2002). Biological reactivity and biomarkers of the neutrophil oxidant, hypochlorous acid. *Toxicology*.

[66] Malle E, Marsche G, Arnhold J, Davies MJ (2006). Modification of low-density lipoprotein by myeloperoxidase-derived oxidants and reagent hypochlorous acid. *Biochimica et Biophysica Acta: Molecular and Cell Biology of Lipids*.

[67] Miyamoto S, Ronsein GE, Prado FM (2007). Biological hydroperoxides and singlet molecular oxygen generation. *IUBMB Life*.

[68] Miyamoto S, Martinez GR, Rettori D, Augusto O, Medeiros MHG, Di Mascio P (2006). Linoleic acid hydroperoxide reacts with hypochlorous acid, generating peroxyl radical intermediates and singlet molecular oxygen. *Proceedings of the National Academy of Sciences of the United States of America*.

[69] Gracanin M, Hawkins CL, Pattison DI, Davies MJ (2009). Singlet-oxygen-mediated amino acid and protein oxidation: formation of tryptophan peroxides and decomposition products. *Free Radical Biology and Medicine*.

[70] Davies MJ (2003). Singlet oxygen-mediated damage to proteins and its consequences. *Biochemical and Biophysical Research Communications*.

[71] Domingues RM, Domingues P, Melo T, Pérez-Sala D, Reis A, Spickett CM (2013). Lipoxidation adducts with peptides and proteins: deleterious modifications or signaling mechanisms?. *Journal of Proteomics*.

[72] Negre-Salvayre A, Coatrieux C, Ingueneau C, Salvayre R (2008). Advanced lipid peroxidation end products in oxidative damage to proteins. Potential role in diseases and therapeutic prospects for the inhibitors. *British Journal of Pharmacology*.

[73] Wang X, Lei XG, Wang J (2014). Malondialdehyde regulates glucose-stimulated insulin secretion in murine islets via TCF7L2-dependent Wnt signaling pathway. *Molecular and Cellular Endocrinology*.

[74] García-Ruiz I, de la Torre P, Díaz T (2002). Sp1 and Sp3 transcription factors mediate malondialdehyde-induced collagen alpha 1(I) gene expression in cultured hepatic stellate cells. *The Journal of Biological Chemistry*.

[75] Li L, Davie JR (2010). The role of Sp1 and Sp3 in normal and cancer cell biology. *Annals of Anatomy: Anatomischer Anzeiger*.

[76] Zarkovic N, Cipak A, Jaganjac M, Borovic S, Zarkovic K (2013). Pathophysiological relevance of aldehydic protein modifications. *Journal of Proteomics*.

[77] Blair IA (2008). DNA adducts with lipid peroxidation products. *Journal of Biological Chemistry*.

[78] Łuczaj W, Skrzydlewska E (2003). DNA damage caused by lipid peroxidation products. *Cellular and Molecular Biology Letters*.

[79] Garcia SC, Grotto D, Bulcão RP (2013). Evaluation of lipid damage related to pathological and physiological conditions. *Drug and Chemical Toxicology*.

[80] Li G, Chen Y, Hu H (2012). Association between age-related decline of kidney function and plasma malondialdehyde. *Rejuvenation Research*.

[81] Sanyal J, Bandyopadhyay SK, Banerjee TK (2009). Plasma levels of lipid peroxides in patients with Parkinson’s disease. *European Review for Medical and Pharmacological Sciences*.

[82] Shanmugam N, Figarola JL, Li Y, Swiderski PM, Rahbar S, Natarajan R (2008). Proinflammatory effects of advanced lipoxidation end products in monocytes. *Diabetes*.

[83] Baskol G, Demir H, Baskol M (2006). Investigation of protein oxidation and lipid peroxidation in patients with rheumatoid arthritis. *Cell Biochemistry and Function*.

[84] Merendino RA, Salvo F, Saija A (2003). Malondialdehyde in benign prostate hypertrophy: a useful marker?. *Mediators of Inflammation*.

[85] Paggiaro PL, Bartoli ML, Novelli F (2011). Malondialdehyde in exhaled breath condensate as a marker of oxidative stress in different pulmonary diseases. *Mediators of Inflammation*.

[86] Hecker M, Ullrich V (1989). On the mechanism of prostacyclin and thromboxane A2 biosynthesis. *Journal of Biological Chemistry*.

[87] Sharma RA, Gescher A, Plastaras JP (2001). Cyclooxygenase-2, malondialdehyde and pyrimidopurinone adducts of deoxyguanosine in human colon cells. *Carcinogenesis*.

[88] Tsikas D, Suchy MT, Niemann J (2012). Glutathione promotes prostaglandin H synthase (cyclooxygenase)-dependent formation of malondialdehyde and 15(S)-8-iso-prostaglandin F2*α*. *FEBS Letters*.

[89] Griesser M, Boeglin WE, Suzuki T, Schneider C (2009). Convergence of the 5-LOX and COX-2 pathways: heme-catalyzed cleavage of the 5S-HETE-derived di-endoperoxide into aldehyde fragments. *Journal of Lipid Research*.

[90] Kadiiska MB, Gladen BC, Baird DD (2005). Biomarkers of oxidative stress study III. Effects of the nonsteroidal anti-inflammatory agents indomethacin and meclofenamic acid on measurements of oxidative products of lipids in CCl4 poisoning. *Free Radical Biology and Medicine*.

[91] Ricciotti E, FitzGerald GA (2011). Prostaglandins and inflammation. *Arteriosclerosis, Thrombosis, and Vascular Biology*.

[92] Ekambaram P, Lambiv W, Cazzolli R, Ashton AW, Honn KV (2011). The thromboxane synthase and receptor signaling pathway in cancer: an emerging paradigm in cancer progression and metastasis. *Cancer and Metastasis Reviews*.

[93] Yang H, Chen C (2008). Cyclooxygenase-2 in synaptic signaling. *Current Pharmaceutical Design*.

[94] Pryor WA, Stanley JP, Blair E (1976). Autoxidation of polyunsaturated fatty acids: II. A suggested mechanism for the formation of TBA reactive materials from prostaglandin like endoperoxides. *Lipids*.

[95] Milne GL, Yin H, Morrow JD (2008). Human biochemistry of the isoprostane pathway. *Journal of Biological Chemistry*.

[96] Gao L, Zackert WE, Hasford JJ (2003). Formation of prostaglandins E2 and D2 via the isoprostane pathway. A mechanism for the generation of bioactive prostaglandins independent of cyclooxygenase. *Journal of Biological Chemistry*.

[97] Yin H, Gao L, Tai H-H, Murphey LJ, Porter NA, Morrow JD (2007). Urinary prostaglandin F2*α* is generated from the isoprostane pathway and not the cyclooxygenase in humans. *Journal of Biological Chemistry*.

[98] Brooks JD, Milne GL, Yin H, Sanchez SC, Porter NA, Morrow JD (2008). Formation of highly reactive cyclopentenone isoprostane compounds (A 3/J3-isoprostanes) in vivo from eicosapentaenoic acid. *Journal of Biological Chemistry*.

[99] Roberts LJ, Fessel JP, Davies SS (2005). The biochemistry of the isoprostane, neuroprostane, and isofuran pathways of lipid peroxidation. *Brain Pathology*.

[100] Onyango AN, Baba N (2010). New hypotheses on the pathways of formation of malondialdehyde and isofurans. *Free Radical Biology and Medicine*.

[101] Siu GM, Draper HH (1982). Metabolism of malonaldehyde in vivo and in vitro. *Lipids*.

[102] Marnett LJ, Buck J, Tuttle MA, Basu AK, Bull AW (1985). Distribution and oxidation of malondialdehyde in mice. *Prostaglandins*.

[103] Agadjanyan ZS, Dmitriev LF, Dugin SF (2005). A new role of phosphoglucose isomerase. Involvement of the glycolytic enzyme in aldehyde metabolism. *Biochemistry*.

[104] Pizzimenti S, Ciamporcero E, Daga M (2013). Interaction of aldehydes derived from lipid peroxidation and membrane proteins. *Frontiers in Physiology*.

[105] Skoumalová A, Hort J (2012). Blood markers of oxidative stress in Alzheimer’s disease. *Journal of Cellular and Molecular Medicine*.

[106] Mangialasche F, Polidori MC, Monastero R (2009). Biomarkers of oxidative and nitrosative damage in Alzheimer’s disease and mild cognitive impairment. *Ageing Research Reviews*.

[107] Pamplona R, Dalfó E, Ayala V (2005). Proteins in human brain cortex are modified by oxidation, glycoxidation, and lipoxidation: effects of Alzheimer disease and identification of lipoxidation targets. *Journal of Biological Chemistry*.

[108] Cristalli DO, Arnal N, Marra FA, De Alaniz MJT, Marra CA (2012). Peripheral markers in neurodegenerative patients and their first-degree relatives. *Journal of the Neurological Sciences*.

[109] Valko M, Leibfritz D, Moncol J, Cronin MTD, Mazur M, Telser J (2007). Free radicals and antioxidants in normal physiological functions and human disease. *International Journal of Biochemistry and Cell Biology*.

[110] López N, Tormo C, De Blas I, Llinares I, Alom J (2013). Oxidative stress in Alzheimer’s disease and mild cognitive impairment with high sensitivity and specificity. *Journal of Alzheimer's Disease*.

[111] Torres LL, Quaglio NB, De Souza GT (2011). Peripheral oxidative stress biomarkers in mild cognitive impairment and alzheimer’s disease. *Journal of Alzheimer’s Disease*.

[112] Polidori MC, Mecocci P (2002). Plasma susceptibility to free radical-induced antioxidant consumption and lipid peroxidation is increased in very old subjects with Alzheimer disease. *Journal of Alzheimer’s Disease*.

[113] Padurariu M, Ciobica A, Hritcu L, Stoica B, Bild W, Stefanescu C (2010). Changes of some oxidative stress markers in the serum of patients with mild cognitive impairment and Alzheimer’s disease. *Neuroscience Letters*.

[114] Sanders LH, Timothy Greenamyre J (2013). Oxidative damage to macromolecules in human Parkinson disease and the rotenone model. *Free Radical Biology and Medicine*.

[115] Mythri RB, Venkateshappa C, Harish G (2011). Evaluation of Markers of oxidative stress, antioxidant function and astrocytic proliferation in the striatum and frontal cortex of Parkinson’s disease brains. *Neurochemical Research*.

[116] Navarro A, Boveris A, Bández MJ (2009). Human brain cortex: mitochondrial oxidative damage and adaptive response in Parkinson disease and in dementia with Lewy bodies. *Free Radical Biology and Medicine*.

[117] Kilinç A, Yalçin AS, Yalçin D, Taga Y, Emerk K (1988). Increased erythrocyte susceptibility to lipid peroxidation in human Parkinson’s disease. *Neuroscience Letters*.

[118] Baillet A, Chanteperdrix V, Trocmé C, Casez P, Garrel C, Besson G (2010). The role of oxidative stress in amyotrophic lateral sclerosis and Parkinson’s disease. *Neurochemical Research*.

[119] Chen CM, Liu JL, Wu YR (2009). Increased oxidative damage in peripheral blood correlates with severity of Parkinson’s disease. *Neurobiology of Disease*.

[120] Kalra J, Rajput AH, Mantha SV, Chaudhary AK, Prasad K (1992). Oxygen free radical producing activity of polymorphonuclear leukocytes in patients with Parkinson’s disease. *Molecular and Cellular Biochemistry*.

[121] Younes-Mhenni S, Frih-Ayed M, Kerkeni A, Bost M, Chazot G (2007). Peripheral blood markers of oxidative stress in Parkinson’s disease. *European Neurology*.

[122] Niedernhofer LJ, Daniels JS, Rouzer CA, Greene RE, Marnett LJ (2003). Malondialdehyde, a product of lipid peroxidation, is mutagenic in human cells. *Journal of Biological Chemistry*.

[123] Del Rio D, Stewart AJ, Pellegrini N (2005). A review of recent studies on malondialdehyde as toxic molecule and biological marker of oxidative stress. *Nutrition, Metabolism and Cardiovascular Diseases*.

[124] VanderVeen LA, Hashim MF, Shyr Y, Marnett LJ (2003). Induction of frameshift and base pair substitution mutations by the major DNA adduct of the endogenous carcinogen malondialdehyde. *Proceedings of the National Academy of Sciences of the United States of America*.

[125] Peluso MEM, Munnia A, Bollati V (2014). Aberrant methylation of hypermethylated-in-cancer-1 and exocyclic DNA adducts in tobacco smokers. *Toxicological Sciences*.

[126] Cai F, Dupertuis YM, Pichard C (2012). Role of polyunsaturated fatty acids and lipid peroxidation on colorectal cancer risk and treatments. *Current Opinion in Clinical Nutrition and Metabolic Care*.

[127] Nair U, Bartsch H, Nair J (2007). Lipid peroxidation-induced DNA damage in cancer-prone inflammatory diseases: a review of published adduct types and levels in humans. *Free Radical Biology and Medicine*.

[128] Bartsch H, Nair J (2005). Accumulation of lipid peroxidation-derived DNA lesions: potential lead markers for chemoprevention of inflammation-driven malignancies. *Mutation Research: Fundamental and Molecular Mechanisms of Mutagenesis*.

[129] Wang M, Dhingra K, Hittelman WN, Liehr JG, De Andrade M, Li D (1996). Lipid peroxidation-induced putative malondialdehyde-DNA adducts in human breast tissues. *Cancer Epidemiology Biomarkers and Prevention*.

[130] Marnett LJ (2012). Inflammation and cancer: chemical approaches to mechanisms, imaging, and treatment. *Journal of Organic Chemistry*.

[131] Dalleau S, Baradat M, Guéraud F, Huc L (2013). Cell death and diseases related to oxidative stress: 4-hydroxynonenal (HNE) in the balance. *Cell Death and Differentiation*.

[132] Barrera G (2012). Oxidative stress and lipid peroxidation products in cancer progression and therapy. *ISRN Oncology*.

[133] Huang H, Kozekov ID, Kozekova A (2010). DNA cross-link induced by trans-4-hydroxynonenal. *Environmental and Molecular Mutagenesis*.

[134] Minko IG, Kozekov ID, Harris TM, Rizzo CJ, Lloyd RS, Stone MP (2009). Chemistry and biology of DNA containing 1,N2-deoxyguanosine adducts of the *α*,*β*-unsaturated aldehydes acrolein, crotonaldehyde, and 4-hydroxynonenal. *Chemical Research in Toxicology*.

[135] Negre-Salvayre A, Auge N, Ayala V (2010). Pathological aspects of lipid peroxidation. *Free Radical Research*.

[136] Chung F-L, Pan J, Choudhury S, Roy R, Hu W, Tang M-S (2003). Formation of trans-4-hydroxy-2-nonenal- and other enal-derived cyclic DNA adducts from *ω*-3 and *ω*-6 polyunsaturated fatty acids and their roles in DNA repair and human p53 gene mutation. *Mutation Research: Fundamental and Molecular Mechanisms of Mutagenesis*.

[137] Lee R, Margaritis M, Channon KM, Antoniades C (2012). Evaluating oxidative stress in human cardiovascular disease: methodological aspects and considerations. *Current Medicinal Chemistry*.

[138] Uchida K (2000). Role of reactive aldehyde in cardiovascular diseases. *Free Radical Biology and Medicine*.

[139] Anderson EJ, Katunga LA, Willis MS (2012). Mitochondria as a source and target of lipid peroxidation products in healthy and diseased heart. *Clinical and Experimental Pharmacology and Physiology*.

[140] Nwose EU, Jelinek HF, Richards RS, Kerr RG (2007). Erythrocyte oxidative stress in clinical management of diabetes and its cardiovascular complications. *British Journal of Biomedical Science*.

[141] Ho E, Karimi Galougahi K, Liu CC, Bhindi R, Figtree GA (2013). Biological markers of oxidative stress: applications to cardiovascular research and practice. *Redox Biology*.

[142] Chapple SJ, Cheng X, Mann GE (2013). Effects of 4-hydroxynonenal on vascular endothelial and smooth muscle cell redox signaling and function in health and disease. *Redox Biology*.

[143] Mattson MP (2009). Roles of the lipid peroxidation product 4-hydroxynonenal in obesity, the metabolic syndrome, and associated vascular and neurodegenerative disorders. *Experimental Gerontology*.

[144] Leonarduzzi G, Chiarpotto E, Biasi F, Poli G (2005). 4-Hydroxynonenal and cholesterol oxidation products in atherosclerosis. *Molecular Nutrition and Food Research*.

[145] Slatter DA, Bolton CH, Bailey AJ (2000). The importance of lipid-derived malondialdehyde in diabetes mellitus. *Diabetologia*.

[146] Bhutia Y, Ghosh A, Sherpa ML, Pal R, Mohanta PK (2011). Serum malondialdehyde level: surrogate stress marker in the Sikkimese diabetics. *Journal of Natural Science, Biology and Medicine*.

[147] Mahreen R, Mohsin M, Nasreen Z, Siraj M, Ishaq M (2010). Significantly increased levels of serum malonaldehyde in type 2 diabetics with myocardial infarction. *International Journal of Diabetes in Developing Countries*.

[148] Tiwari BK, Pandey KB, Abidi AB, Rizvi SI (2013). Markers of oxidative stress during diabetes mellitus. *Journal of Biomarkers*.

[149] Nakhjavani M, Esteghamati A, Nowroozi S, Asgarani F, Rashidi A, Khalilzadeh O (2010). Type 2 diabetes mellitus duration: an independent predictor of serum malondialdehyde levels. *Singapore Medical Journal*.

[150] Wang CH, Chang RW, Ko YH (2014). Prevention of arterial stiffening by using low-dose atorvastatin in diabetes is associated with decreased malondialdehyde. *PloS ONE*.

[151] Jaganjac M, Tirosh O, Cohen G, Sasson S, Zarkovic N (2013). Reactive aldehydes—second messengers of free radicals in diabetes mellitus. *Free Radical Research*.

[152] Cohen G, Riahi Y, Shamni O (2011). Role of lipid peroxidation and PPAR-*δ* in amplifying glucose-stimulated insulin secretion. *Diabetes*.

[153] Pradeep AR, Agarwal E, Bajaj P, Rao NS (2013). 4-Hydroxy-2-nonenal, an oxidative stress marker in crevicular fluid and serum in type 2 diabetes with chronic periodontitis. *Contemporary Clinical Dentistry*.

[154] Toyokuni S, Yamada S, Kashima M (2000). Serum 4-hydroxy-2-nonenal-modified albumin is elevated in patients with type 2 diabetes mellitus. *Antioxidants and Redox Signaling*.

[155] Cohen G, Riahi Y, Sunda V (2013). Signaling properties of 4-hydroxyalkenals formed by lipid peroxidation in diabetes. *Free Radical Biology and Medicine*.

[156] Lupachyk S, Shevalye H, Maksimchyk Y, Drel VR, Obrosova IG (2011). PARP inhibition alleviates diabetes-induced systemic oxidative stress and neural tissue 4-hydroxynonenal adduct accumulation: correlation with peripheral nerve function. *Free Radical Biology and Medicine*.

[157] Tuma DJ (2002). Role of malondialdehyde-acetaldehyde adducts in liver injury. *Free Radical Biology and Medicine*.

[158] Sampey BP, Korourian S, Ronis MJ, Badger TM, Petersen DR (2003). Immunohistochemical characterization of hepatic malondialdehyde and 4-hydroxynonenal modified proteins during early stages of ethanol-induced liver injury. *Alcoholism: Clinical and Experimental Research*.

[159] Albano E (2012). Role of adaptive immunity in alcoholic liver disease. *International Journal of Hepatology*.

[160] Thiele GM, Klassen LW, Tuma DJ (2008). Formation and immunological properties of aldehyde-derived protein adducts following alcohol consumption. *Methods in Molecular Biology*.

[161] Das SK, Vasudevan DM (2007). Alcohol-induced oxidative stress. *Life Sciences*.

[162] Niemelä O (2001). Distribution of ethanol-induced protein adducts in vivo: relationship to tissue injury. *Free Radical Biology and Medicine*.

[163] Mottaran E, Stewart SF, Rolla R (2002). Lipid peroxidation contributes to immune reactions associated with alcoholic liver disease. *Free Radical Biology and Medicine*.

[164] Willis MS, Klassen LW, Tuma DJ, Sorrell MF, Thiele GM (2002). Adduction of soluble proteins with malondialdehyde-acetaldehyde (MAA) induces antibody production and enhances T-cell proliferation. *Alcoholism: Clinical and Experimental Research*.

[165] Dou X, Li S, Wang Z (2012). Inhibition of NF-*κ*B activation by 4-hydroxynonenal contributes to liver injury in a mouse model of alcoholic liver disease. *The American Journal of Pathology*.

[166] Smathers RL, Galligan JJ, Stewart BJ, Petersen DR (2011). Overview of lipid peroxidation products and hepatic protein modification in alcoholic liver disease. *Chemico-Biological Interactions*.

[167] Poli G, Biasi F, Leonarduzzi G (2008). 4-Hydroxynonenal-protein adducts: a reliable biomarker of lipid oxidation in liver diseases. *Molecular Aspects of Medicine*.

[168] Petersen DR, Doorn JA (2004). Reactions of 4-hydroxynonenal with proteins and cellular targets. *Free Radical Biology and Medicine*.

[169] Galligan JJ, Smathers RL, Fritz KS, Epperson LE, Hunter LE, Petersen DR (2012). Protein carbonylation in a murine model for early alcoholic liver disease. *Chemical Research in Toxicology*.

[170] Perluigi M, Coccia R, Butterfield DA (2012). 4-Hydroxy-2-nonenal, a reactive product of lipid peroxidation, and neurodegenerative diseases: a toxic combination illuminated by redox proteomics studies. *Antioxidants & Redox Signaling*.

[171] Reed TT (2011). Lipid peroxidation and neurodegenerative disease. *Free Radical Biology and Medicine*.

[172] Zarkovic K (2003). 4-hydroxynonenal and neurodegenerative diseases. *Molecular Aspects of Medicine*.

[173] Selley ML (1998). (E)-4-hydroxy-2-nonenal may be involved in the pathogenesis of Parkinson’s disease. *Free Radical Biology and Medicine*.

[174] Yoritaka A, Hattori N, Uchida K, Tanaka M, Stadtman ER, Mizuno Y (1996). Immunohistochemical detection of 4-hydroxynonenal protein adducts in Parkinson disease. *Proceedings of the National Academy of Sciences of the United States of America*.

[175] Traverso N, Menini S, Maineri EP (2004). Malondialdehyde, a lipoperoxidation-derived aldehyde, can bring about secondary oxidative damage to proteins. *Journals of Gerontology A: Biological Sciences and Medical Sciences*.

[176] Tuma DJ, Kearley ML, Thiele GM (2001). Elucidation of reaction scheme describing malondialdehyde—acetaldehyde—protein adduct formation. *Chemical Research in Toxicology*.

[177] Wang G, Li H, Firoze Khan M (2012). Differential oxidative modification of proteins in MRL+/+ and MRL/lpr mice: increased formation of lipid peroxidation-derived aldehyde-protein adducts may contribute to accelerated onset of autoimmune response. *Free Radical Research*.

[178] Duryee MJ, Klassen LW, Jones BL, Willis MS, Tuma DJ, Thiele GM (2008). Increased immunogenicity to P815 cells modified with malondialdehyde and acetaldehyde. *International Immunopharmacology*.

[179] Wang G, Ansari GAS, Khan MF (2007). Involvement of lipid peroxidation-derived aldehyde-protein adducts in autoimmunity mediated by trichloroethene. *Journal of Toxicology and Environmental Health A: Current Issues*.

[180] Wållberg M, Bergquist J, Achour A, Breij E, Harris RA (2007). Malondialdehyde modification of myelin oligodendrocyte glycoprotein leads to increased immunogenicity and encephalitogenicity. *European Journal of Immunology*.

[181] Weismann D, Binder CJ (2012). The innate immune response to products of phospholipid peroxidation. *Biochimica et Biophysica Acta: Biomembranes*.

[182] Slatter DA, Avery NC, Bailey AJ (2004). Identification of a new cross-link and unique histidine adduct from bovine serum albumin incubated with malondialdehyde. *Journal of Biological Chemistry*.

[183] Cheng J, Wang F, Yu D-F, Wu P-F, Chen J-G (2011). The cytotoxic mechanism of malondialdehyde and protective effect of carnosine via protein cross-linking/mitochondrial dysfunction/reactive oxygen species/MAPK pathway in neurons. *European Journal of Pharmacology*.

[184] Weismann D, Hartvigsen K, Lauer N (2011). Complement factor H binds malondialdehyde epitopes and protects from oxidative stress. *Nature*.

[185] Veneskoski M, Turunen SP, Kummu O (2011). Specific recognition of malondialdehyde and malondialdehyde acetaldehyde adducts on oxidized LDL and apoptotic cells by complement anaphylatoxin C3a. *Free Radical Biology and Medicine*.

[186] Kharbanda KK, Shubert KA, Wyatt TA, Sorrell MF, Tuma DJ (2002). Effect of malondialdehyde-acetaldehyde-protein adducts on the protein kinase C-dependent secretion of urokinase-type plasminogen activator in hepatic stellate cells. *Biochemical Pharmacology*.

[187] Marnett LJ (2002). Oxy radicals, lipid peroxidation and DNA damage. *Toxicology*.

[188] Marnett LJ (1999). Lipid peroxidation-DNA damage by malondialdehyde. *Mutation Research*.

[189] Fink SP, Reddy GR, Marnett LJ (1997). Mutagenicity in Escherichia coli of the major DNA adduct derived from the endogenous mutagen malondialdehyde. *Proceedings of the National Academy of Sciences of the United States of America*.

[190] Vöhringer M-L, Becker TW, Krieger G, Jacobi H, Witte I (1998). Synergistic DNA damaging effects of malondialdehyde/Cu(II) in PM2 DNA and in human fibroblasts. *Toxicology Letters*.

[191] Ji C, Rouzer CA, Marnett LJ, Pietenpol JA (1998). Induction of cell cycle arrest by the endogenous product of lipid peroxidation, malondialdehyde. *Carcinogenesis*.

[192] Willis MS, Klassen LW, Carlson DL, Brouse CF, Thiele GM (2004). Malondialdehyde-acetaldehyde haptenated protein binds macrophage scavenger receptor(s) and induces lysosomal damage. *International Immunopharmacology*.

[193] Otteneder MB, Knutson CG, Daniels JS (2006). In vivo oxidative metabolism of a major peroxidation-derived DNA adduct, M1dG. *Proceedings of the National Academy of Sciences of the United States of America*.

[194] Cline SD, Lodeiro MF, Marnett LJ, Cameron CE, Arnold JJ (2010). Arrest of human mitochondrial RNA polymerase transcription by the biological aldehyde adduct of DNA, M1dG. *Nucleic Acids Research*.

[195] Sram RJ, Farmer P, Singh R (2009). Effect of vitamin levels on biomarkers of exposure and oxidative damage-the EXPAH study. *Mutation Research: Genetic Toxicology and Environmental Mutagenesis*.

[196] Spickett CM (2013). The lipid peroxidation product 4-hydroxy-2-nonenal: advances in chemistry and analysis. *Redox Biology*.

[197] Usatyuk PV, Natarajan V (2012). Hydroxyalkenals and oxidized phospholipids modulation of endothelial cytoskeleton, focal adhesion and adherens junction proteins in regulating endothelial barrier function. *Microvascular Research*.

[198] Sharma R, Sharma A, Chaudhary P (2012). Role of 4-hydroxynonenal in chemopreventive activities of sulforaphane. *Free Radical Biology and Medicine*.

[199] Zimniak P (2011). Relationship of electrophilic stress to aging. *Free Radical Biology and Medicine*.

[200] Fritz KS, Petersen DR (2011). Exploring the biology of lipid peroxidation-derived protein carbonylation. *Chemical Research in Toxicology*.

[201] Butterfield DA, Reed T, Sultana R (2011). Roles of 3-nitrotyrosine- and 4-hydroxynonenal-modified brain proteins in the progression and pathogenesis of Alzheimer’s disease. *Free Radical Research*.

[202] Balogh LM, Atkins WM (2011). Interactions of glutathione transferases with 4-hydroxynonenal. *Drug Metabolism Reviews*.

[203] Klil-Drori AJ, Ariel A (2013). 15-Lipoxygenases in cancer: a double-edged sword?. *Prostaglandins & Other Lipid Mediators*.

[204] Brash AR, Boeglin WE, Chang MS (1997). Discovery of a second 15S-lipoxygenase in humans. *Proceedings of the National Academy of Sciences of the United States of America*.

[205] Ivanov I, Heydeck D, Hofheinz K (2010). Molecular enzymology of lipoxygenases. *Archives of Biochemistry and Biophysics*.

[206] Takamura H, Gardner HW (1996). Oxygenation of (3Z)-alkenal to (2E)-4-hydroxy-2-alkenal in soybean seed (Glycine max L.). *Biochimica et Biophysica Acta: Lipids and Lipid Metabolism*.

[207] Schneider C, Tallman KA, Porter NA, Brash AR (2001). Two distinct pathways of formation of 4-hydroxynonenal. Mechanisms of nonenzymatic transformation of the 9- and 13-hydroperoxides of linoleic acid to 4-hydroxyalkenals. *Journal of Biological Chemistry*.

[208] Riahi Y, Cohen G, Shamni O, Sasson S (2010). Signaling and cytotoxic functions of 4-hydroxyalkenals. *American Journal of Physiology: Endocrinology and Metabolism*.

[209] Mahipal SVK, Subhashini J, Reddy MC (2007). Effect of 15-lipoxygenase metabolites, 15-(S)-HPETE and 15-(S)-HETE on chronic myelogenous leukemia cell line K-562: reactive oxygen species (ROS) mediate caspase-dependent apoptosis. *Biochemical Pharmacology*.

[210] Kumar KA, Arunasree KM, Roy KR (2009). Effects of (15S)-hydroperoxyeicosatetraenoic acid and (15S)-hydroxyeicosatetraenoic acid on the acute-lymphoblastic-leukaemia cell line Jurkat: activation of the Fas-mediated death pathway. *Biotechnology and Applied Biochemistry*.

[211] Eckl PM (2003). Genotoxicity of HNE. *Molecular Aspects of Medicine*.

[212] Siems W, Grune T (2003). Intracellular metabolism of 4-hydroxynonenal. *Molecular Aspects of Medicine*.

[213] Alary J, Guéraud F, Cravedi J-P (2003). Fate of 4-hydroxynonenal in vivo: disposition and metabolic pathways. *Molecular Aspects of Medicine*.

[214] McElhanon KE, Bose C, Sharma R, Wu L, Awasthi YC, Singh SP (2013). Gsta4 null mouse embryonic fibroblasts exhibit enhanced sensitivity to oxidants: role of 4-hydroxynonenal in oxidant toxicity. *Open Journal of Apoptosis*.

[215] Black W, Chen Y, Matsumoto A (2012). Molecular mechanisms of ALDH3A1-mediated cellular protection against 4-hydroxy-2-nonenal. *Free Radical Biology and Medicine*.

[216] Kong D, Kotraiah V (2012). Modulation of aldehyde dehydrogenase activity affects (±)-4-hydroxy-2E-nonenal (HNE) toxicity and HNE-protein adduct levels in PC12 cells. *Journal of Molecular Neuroscience*.

[217] Huang Y, Li W, Kong ANT (2012). Anti-oxidative stress regulator NF-E2-related factor 2 mediates the adaptive induction of antioxidant and detoxifying enzymes by lipid peroxidation metabolite 4-hydroxynonenal. *Cell & Bioscience*.

[218] Zhang Y, Sano M, Shinmura K (2010). 4-Hydroxy-2-nonenal protects against cardiac ischemia-reperfusion injury via the Nrf2-dependent pathway. *Journal of Molecular and Cellular Cardiology*.

[219] Siow RCM, Ishii T, Mann GE (2007). Modulation of antioxidant gene expression by 4-hydroxynonenal: atheroprotective role of the Nrf2/ARE transcription pathway. *Redox Report*.

[220] Tanito M, Agbaga M-P, Anderson RE (2007). Upregulation of thioredoxin system via Nrf2-antioxidant responsive element pathway in adaptive-retinal neuroprotection in vivo and in vitro. *Free Radical Biology and Medicine*.

[221] Ishii T, Itoh K, Ruiz E (2004). Role of Nrf2 in the regulation of CD36 and stress protein expression in murine macrophages: activation by oxidatively modified LDL and 4-hydroxynonenal. *Circulation Research*.

[222] Miller DM, Singh IN, Wang JA, Hall ED (2013). Administration of the Nrf2-ARE activators sulforaphane and carnosic acid attenuates 4-hydroxy-2-nonenal-induced mitochondrial dysfunction ex vivo. *Free Radical Biology and Medicine*.

[223] Gan L, Johnson JA (2013). Oxidative damage and the Nrf2-ARE pathway in neurodegenerative diseases. *Biochimica et Biophysica Acta: Molecular Basis of Disease*.

[224] Na HK, Surh YJ (2014). Oncogenic potential of Nrf2 and its principal target protein heme oxygenase-1. *Free Radical Biology and Medicine*.

[225] Seo HA, Lee IK (2013). The role of Nrf2: adipocyte differentiation, obesity, and insulin resistance. *Oxidative Medicine and Cellular Longevity*.

[226] Deramaudt TB, Dill C, Bonay M (2013). Regulation of oxidative stress by Nrf2 in the pathophysiology of infectious diseases. *Médecine et Maladies Infectieuses*.

[227] Grochot-Przeczek A, Dulak J, Jozkowicz A (2012). Haem oxygenase-1: non-canonical roles in physiology and pathology. *Clinical Science*.

[228] Lin MH, Yen JH, Weng CY, Wang L, Ha CL, Wu MJ (2014). Lipid peroxidation end product 4-hydroxy-trans-2-nonenal triggers unfolded protein response and heme oxygenase-1 expression in PC12 cells: roles of ROS and MAPK pathways. *Toxicology*.

[229] Ishikado A, Nishio Y, Morino K (2010). Low concentration of 4-hydroxy hexenal increases heme oxygenase-1 expression through activation of Nrf2 and antioxidative activity in vascular endothelial cells. *Biochemical and Biophysical Research Communications*.

[230] Ueda K, Ueyama T, Yoshida K-I (2008). Adaptive HNE-Nrf2-HO-1 pathway against oxidative stress is associated with acute gastric mucosal lesions. *American Journal of Physiology: Gastrointestinal and Liver Physiology*.

[231] Holmgren A, Lu J (2010). Thioredoxin and thioredoxin reductase: current research with special reference to human disease. *Biochemical and Biophysical Research Communications*.

[232] Chen Z-H, Saito Y, Yoshida Y, Sekine A, Noguchi N, Niki E (2005). 4-hydroxynonenal induces adaptive response and enhances PC12 cell tolerance primarily through induction of thioredoxin reductase 1 via activation of Nrf2. *Journal of Biological Chemistry*.

[233] Lu SC (2013). Glutathione synthesis. *Biochimica et Biophysica Acta*.

[234] Franklin CC, Backos DS, Mohar I, White CC, Forman HJ, Kavanagh TJ (2009). Structure, function, and post-translational regulation of the catalytic and modifier subunits of glutamate cysteine ligase. *Molecular Aspects of Medicine*.

[235] Backos DS, Fritz KS, Roede JR, Petersen DR, Franklin CC (2011). Posttranslational modification and regulation of glutamate-cysteine ligase by the *α*,*β*-unsaturated aldehyde 4-hydroxy-2-nonenal. *Free Radical Biology and Medicine*.

[236] Zhang H, Shih A, Rinna A, Forman HJ (2009). Resveratrol and 4-hydroxynonenal act in concert to increase glutamate cysteine ligase expression and glutathione in human bronchial epithelial cells. *Archives of Biochemistry and Biophysics*.

[237] Zhang H, Court N, Forman HJ (2007). Submicromolar concentrations of 4-hydroxynonenal induce glutamate cysteine ligase expression in HBE1 cells. *Redox Report*.

[238] Iles KE, Liu R-M (2005). Mechanisms of Glutamate Cysteine Ligase (GCL) induction by 4-hydroxynonenal. *Free Radical Biology and Medicine*.

[239] Forman HJ, Dickinson DA, Iles KE (2003). HNE—signaling pathways leading to its elimination. *Molecular Aspects of Medicine*.

[240] Braithwaite EK, Mattie MD, Freedman JH (2010). Activation of metallothionein transcription by 4-hydroxynonenal. *Journal of Biochemical and Molecular Toxicology*.

[241] Reichard JF, Petersen DR (2004). Hepatic stellate cells lack AP-1 responsiveness to electrophiles and phorbol 12-myristate-13-acetate. *Biochemical and Biophysical Research Communications*.

[242] Kikuta K, Masamune A, Satoh M, Suzuki N, Shimosegawa T (2004). 4-Hydroxy-2, 3-nonenal activates activator protein-1 and mitogen-activated protein kinases in rat pancreatic stellate cells. *World Journal of Gastroenterology*.

[243] Camandola S, Poli G, Mattson MP (2000). The lipid peroxidation product 4-hydroxy-2,3-nonenal increases AP-1- binding activity through caspase activation in neurons. *Journal of Neurochemistry*.

[244] Shaulian E, Karin M (2002). AP-1 as a regulator of cell life and death. *Nature Cell Biology*.

[245] Shaulian E (2010). AP-1—the Jun proteins: oncogenes or tumor suppressors in disguise?. *Cellular Signalling*.

[246] Morgan MJ, Liu Z (2011). Crosstalk of reactive oxygen species and NF-*κ*B signaling. *Cell Research*.

[247] Siomek A (2012). NF-*κ*B signaling pathway and free radical impact. *Acta Biochimica Polonica*.

[248] Lim JH, Lee J-C, Lee YH (2006). Simvastatin prevents oxygen and glucose deprivation/reoxygenation-induced death of cortical neurons by reducing the production and toxicity of 4-hydroxy-2E-nonenal. *Journal of Neurochemistry*.

[249] Kaarniranta K, Ryhänen T, Karjalainen HM (2005). Geldanamycin increases 4-hydroxynonenal (HNE)-induced cell death in human retinal pigment epithelial cells. *Neuroscience Letters*.

[250] Luckey SW, Taylor M, Sampey BP, Scheinman RI, Petersen DR (2002). 4-Hydroxynonenal decreases interleukin-6 expression and protein production in primary rat Kupffer cells by inhibiting nuclear factor-*κ*B activation. *Journal of Pharmacology and Experimental Therapeutics*.

[251] Minekura H, Kumagai T, Kawamoto Y, Nara F, Uchida K (2001). 4-Hydroxy-2-nonenal is a powerful endogenous inhibitor of endothelial response. *Biochemical and Biophysical Research Communications*.

[252] Ji C, Kozak KR, Marnett LJ (2001). I*κ*B kinase, a molecular target for inhibition by 4-hydroxy-2-nonenal. *Journal of Biological Chemistry*.

[253] Lee SJ, Kim CE, Seo KW, Kim CD (2010). HNE-induced 5-LO expression is regulated by NF-*κ*B/ERK and Sp1/p38 MAPK pathways via EGF receptor in murine macrophages. *Cardiovascular Research*.

[254] Lee SJ, Seo KW, Yun MR (2008). 4-hydroxynonenal enhances MMP-2 production in vascular smooth muscle cells via mitochondrial ROS-mediated activation of the Akt/NF-*κ*B signaling pathways. *Free Radical Biology and Medicine*.

[255] Raza H, John A, Brown EM, Benedict S, Kambal A (2008). Alterations in mitochondrial respiratory functions, redox metabolism and apoptosis by oxidant 4-hydroxynonenal and antioxidants curcumin and melatonin in PC12 cells. *Toxicology and Applied Pharmacology*.

[256] Malone PE, Hernandez MR (2007). 4-Hydroxynonenal, a product of oxidative stress, leads to an antioxidant response in optic nerve head astrocytes. *Experimental Eye Research*.

[257] Vaillancourt F, Morquette B, Shi Q (2007). Differential regulation of cyclooxygenase-2 and inducible nitric oxide synthase by 4-hydroxynonenal in human osteoarthritic chondrocytes through ATF-2/CREB-1 transactivation and concomitant inhibition of NF-*κ*B signaling cascade. *Journal of Cellular Biochemistry*.

[258] Amma H, Naruse K, Ishiguro N, Sokabe M (2005). Involvement of reactive oxygen species in cyclic stretch-induced NF-*κ*B activation in human fibroblast cells. *British Journal of Pharmacology*.

[259] Donath B, Fischer C, Page S (2002). Chlamydia pneumoniae activates IKK/I*κ*B-mediated signaling, which is inhibited by 4-HNE and following primary exposure. *Atherosclerosis*.

[260] Kim T, Yang Q (2013). Peroxisome-proliferator-activated receptors regulate redox signaling in the cardiovascular system. *World Journal of Cardiology*.

[261] Ahmadian M, Suh JM, Hah N (2013). PPAR*γ* signaling and metabolism: the good, the bad and the future. *Nature Medicine*.

[262] Barrera G, Toaldo C, Pizzimenti S (2008). The role of PPAR ligands in controlling growth-related gene expression and their interaction with lipoperoxidation products. *PPAR Research*.

[263] Wang Z, Dou X, Gu D (2012). 4-Hydroxynonenal differentially regulates adiponectin gene expression and secretion via activating PPAR*γ* and accelerating ubiquitin-proteasome degradation. *Molecular and Cellular Endocrinology*.

[264] Pizzimenti S, Laurora S, Briatore F, Ferretti C, Dianzani MU, Barrera G (2002). Synergistic effect of 4-hydroxynonenal and PPAR ligands in controlling human leukemic cell growth and differentiation. *Free Radical Biology and Medicine*.

[265] Cerbone A, Toaldo C, Laurora S (2007). 4-hydroxynonenal and PPAR*γ* ligands affect proliferation, differentiation, and apoptosis in colon cancer cells. *Free Radical Biology and Medicine*.

[266] Almeida M, Ambrogini E, Han L, Manolagas SC, Jilka RL (2009). Increased lipid oxidation causes oxidative stress, increased peroxisome proliferator-activated receptor-*γ* expression, and diminished pro-osteogenic Wnt signaling in the skeleton. *Journal of Biological Chemistry*.

[267] Coleman JD, Prabhu KS, Thompson JT (2007). The oxidative stress mediator 4-hydroxynonenal is an intracellular agonist of the nuclear receptor peroxisome proliferator-activated receptor-*β*/*δ* (PPAR*β*/*δ*). *Free Radical Biology and Medicine*.

[268] Zheng R, Po I, Mishin V (2013). The generation of 4-hydroxynonenal, an electrophilic lipid peroxidation end product, in rabbit cornea organ cultures treated with UVB light and nitrogen mustard. *Toxicology and Applied Pharmacology*.

[269] Zheng R, Heck DE, Mishin V (2014). Modulation of keratinocyte expression of antioxidants by 4-hydroxynonenal, a lipid peroxidation end product. *Toxicology and Applied Pharmacology*.

[270] Uchida K, Kumagai T (2003). 4-Hydroxy-2-nonenal as a COX-2 inducer. *Molecular Aspects of Medicine*.

[271] Parola M, Robino G, Marra F (1998). HNE interacts directly with JNK isoforms in human hepatic stellate cells. *Journal of Clinical Investigation*.

[272] Rinna A, Forman HJ (2008). SHP-1 inhibition by 4-hydroxynonenal activates Jun N-terminal kinase and glutamate cysteine ligase. *American Journal of Respiratory Cell and Molecular Biology*.

[273] Liu R-M, Borok Z, Forman HJ (2001). 4-Hydroxy-2-nonenal increases *γ*-glutamylcysteine synthetase gene expression in alveolar epithelial cells. *American Journal of Respiratory Cell and Molecular Biology*.

[274] Dickinson DA, Iles KE, Watanabe N (2002). 4-Hydroxynonenal induces glutamate cysteine ligase through JNK in HBE1 cells. *Free Radical Biology and Medicine*.

[275] Marantos C, Mukaro V, Ferrante J, Hii C, Ferrante A (2008). Inhibition of the lipopolysaccharide-induced stimulation of the members of the MAPK family in human monocytes/macrophages by 4-hydroxynonenal, a product of oxidized omega-6 fatty acids. *American Journal of Pathology*.

[276] Shi Q, Vaillancourt F, Côté V (2006). Alterations of metabolic activity in human osteoarthritic osteoblasts by lipid peroxidation end product 4-hydroxynonenal. *Arthritis Research and Therapy*.

[277] Usatyuk PV, Parinandi NL, Natarajan V (2006). Redox regulation of 4-hydroxy-2-nonenal-mediated endothelial barrier dysfunction by focal adhesion, adherens, and tight junction proteins. *Journal of Biological Chemistry*.

[278] Shibata N, Kato Y, Inose Y (2011). 4-hydroxy-2-nonenal upregulates and phosphorylates cytosolic phospholipase A_2_ in cultured Ra2 microglial cells via MAPK pathways. *Neuropathology*.

[279] Verslegers M, Lemmens K, Van Hove I, Moons L (2013). Matrix metalloproteinase-2 and -9 as promising benefactors in development, plasticity and repair of the nervous system. *Progress in Neurobiology*.

[280] Lee SJ, Kim CE, Yun MR (2010). 4-Hydroxynonenal enhances MMP-9 production in murine macrophages via 5-lipoxygenase-mediated activation of ERK and p38 MAPK. *Toxicology and Applied Pharmacology*.

[281] Seo KW, Lee SJ, Kim CE (2010). Participation of 5-lipoxygenase-derived LTB4 in 4-hydroxynonenal-enhanced MMP-2 production in vascular smooth muscle cells. *Atherosclerosis*.

[282] Morquette B, Shi Q, Lavigne P, Ranger P, Fernandes JC, Benderdour M (2006). Production of lipid peroxidation products in osteoarthritic tissues: new evidence linking 4-hydroxynonenal to cartilage degradation. *Arthritis and Rheumatism*.

[283] Hers I, Vincent EE, Tavaré JM (2011). Akt signalling in health and disease. *Cell Signaling*.

[284] Chalhoub N, Baker SJ (2009). PTEN and the PI3-kinase pathway in cancer. *Annual Review of Pathology*.

[285] Shearn CT, Fritz KS, Reigan P, Petersen DR (2011). Modification of Akt2 by 4-hydroxynonenal inhibits insulin-dependent Akt signaling in HepG2 cells. *Biochemistry*.

[286] Shearn CT, Smathers RL, Backos DS, Reigan P, Orlicky DJ, Petersen DR (2013). Increased carbonylation of the lipid phosphatase PTEN contributes to Akt2 activation in a murine model of early alcohol-induced steatosis. *Free Radical Biology and Medicine*.

[287] Shearn CT, Reigan P, Petersen DR (2012). Inhibition of Hydrogen peroxide signaling by 4-hydroxynonenal due to differential regulation of Akt1 and Akt2 contributes to decreases in cell survival and proliferation in hepatocellular carcinoma cells. *Free Radical Biology and Medicine*.

[288] Vatsyayan R, Chaudhary P, Sharma A (2011). Role of 4-hydroxynonenal in epidermal growth factor receptor-mediated signaling in retinal pigment epithelial cells. *Experimental Eye Research*.

[289] Turban S, Hajduch E (2011). Protein kinase C isoforms: mediators of reactive lipid metabolites in the development of insulin resistance. *FEBS Letters*.

[290] Maggiora M, Rossi MA (2006). The exocytosis induced in HL-60 cells by 4-hydroxynonenal, a lipid peroxidation product, is not prevented by reduced glutathione. *Cell Biochemistry and Function*.

[291] Maggiora M, Rossi MA (2003). Experimental researches on the role of phosphoinositide-specific phospholipase C in 4-hydroxynonenal induced exocytosis. *Cell Biochemistry and Function*.

[292] Rossi MA, Di Mauro C, Dianzani MU (2001). Experimental studies on the mechanism of phospholipase C activation by the lipid peroxidation products 4-hydroxynonenal and 2-nonenal. *International Journal of Tissue Reactions*.

[293] Rossi MA, Di Mauro C, Esterbauer H, Fidale F, Dianzani MU (1994). Activation of phosphoinositide-specific phospholipase C of rat neutrophils by the chemotactic aldehydes 4-hydroxy-2,3-trans-nonenal and 4-hydroxy-2,3-trans-octenal. *Cell Biochemistry and Function*.

[294] de Oliveira-Junior EB, Bustamante J, Newburger PE, Condino-Neto A (2011). The human NADPH oxidase: primary and secondary defects impairing the respiratory burst function and the microbicidal ability of phagocytes. *Scandinavian Journal of Immunology*.

[295] Harry RS, Hiatt LA, Kimmel DW (2012). Metabolic impact of 4-hydroxynonenal on macrophage-like RAW 264.7 function and activation. *Chemical Research in Toxicology*.

[296] Chiarpotto E, Domenicotti C, Paola D (1999). Regulation of rat hepatocyte protein kinase C *β* isoenzymes by the lipid peroxidation product 4-hydroxy-2,3-nonenal: a signaling pathway to modulate vesicular transport of glycoproteins. *Hepatology*.

[297] Paola D, Domenicotti C, Nitti M (2000). Oxidative stress induces increase in intracellular amyloid *β*-protein production and selective activation of *β*I and *β*II PKCs in NT2 cells. *Biochemical and Biophysical Research Communications*.

[298] Marinari UM, Nitti M, Pronzato MA, Domenicotti C (2003). Role of PKC-dependent pathways in HNE-induced cell protein transport and secretion. *Molecular Aspects of Medicine*.

[299] Nitti M, Domenicotti C, D’Abramo C (2002). Activation of PKC-*β* isoforms mediates HNE-induced MCP-1 release by macrophages. *Biochemical and Biophysical Research Communications*.

[300] Ramana KV, Fadl AA, Tammali R, Reddy ABM, Chopra AK, Srivastava SK (2006). Aldose reductase mediates the lipopolysaccharide-induced release of inflammatory mediators in RAW264.7 murine macrophages. *Journal of Biological Chemistry*.

[301] Dodson M, Darley-Usmar V, Zhang J (2013). Cellular metabolic and autophagic pathways: traffic control by redox signaling. *Free Radical Biology and Medicine*.

[302] Hill BG, Haberzettl P, Ahmed Y, Srivastava S, Bhatnagar A (2008). Unsaturated lipid peroxidation-derived aldehydes activate autophagy in vascular smooth-muscle cells. *Biochemical Journal*.

[303] Haberzettl P, Hill BG (2013). Oxidized lipids activate autophagy in a JNK-dependent manner by stimulating the endoplasmic reticulum stress response. *Redox Biology*.

[304] Dodson M, Liang Q, Johnson MS (2013). Inhibition of glycolysis attenuates 4-hydroxynonenal-dependent autophagy and exacerbates apoptosis in differentiated SH-SY5Y neuroblastoma cells. *Autophagy*.

[305] Krohne TU, Stratmann NK, Kopitz J, Holz FG (2010). Effects of lipid peroxidation products on lipofuscinogenesis and autophagy in human retinal pigment epithelial cells. *Experimental Eye Research*.

[306] Fyhrquist F, Saijonmaa O, Strandberg T (2013). The roles of senescence and telomere shortening in cardiovascular disease. *Nature Reviews Cardiology*.

[307] Olive PL (2009). Endogenous DNA breaks: gammaH2AX and the role of telomeres. *Aging*.

[308] Günes C, Rudolph KL (2013). The role of telomeres in stem cells and cancer. *Cell*.

[309] Argüelles S, Machado A, Ayala A (2009). Adduct formation of 4-hydroxynonenal and malondialdehyde with elongation factor-2 in vitro and in vivo. *Free Radical Biology and Medicine*.

[310] Wang C, Maddick M, Miwa S (2010). Adult-onset, short-term dietary restriction reduces cell senescence in mice. *Aging*.

[311] Nelson G, Wordsworth J, Wang C (2012). A senescent cell bystander effect: senescence-induced senescence. *Aging Cell*.

[312] Voghel G, Thorin-Trescases N, Farhat N (2008). Chronic treatment with N-acetyl-cystein delays cellular senescence in endothelial cells isolated from a subgroup of atherosclerotic patients. *Mechanisms of Ageing and Development*.

[313] Pizzimenti S, Briatore F, Laurora S (2006). 4-Hydroxynonenal inhibits telomerase activity and hTERT expression in human leukemic cell lines. *Free Radical Biology and Medicine*.

[314] Pizzimenti S, Menegatti E, Berardi D (2010). 4-Hydroxynonenal, a lipid peroxidation product of dietary polyunsaturated fatty acids, has anticarcinogenic properties in colon carcinoma cell lines through the inhibition of telomerase activity. *Journal of Nutritional Biochemistry*.

[315] Rufini A, Tucci P, Celardo I, Melino G (2013). Senescence and aging: the critical roles of p53. *Oncogene*.

[316] Qian Y, Chen X (2013). Senescence regulation by the p53 protein family. *Methods in Molecular Biology*.

[317] Sahin E, DePinho RA (2012). Axis of ageing: telomeres, p53 and mitochondria. *Nature Reviews Molecular Cell Biology*.

[318] Liu D, Xu Y (2011). P53, oxidative stress, and aging. *Antioxidants and Redox Signaling*.

[319] Hafsi H, Hainaut P (2011). Redox control and interplay between p53 isoforms: roles in the regulation of basal p53 levels, cell fate, and senescence. *Antioxidants and Redox Signaling*.

[320] Vigneron A, Vousden KH (2010). p53, ROS and senescence in the control of aging. *Aging*.

[321] Verbon EH, Post JA, Boonstra J (2012). The influence of reactive oxygen species on cell cycle progression in mammalian cells. *Gene*.

[322] Chiu J, Dawes IW (2012). Redox control of cell proliferation. *Trends in Cell Biology*.

[323] Lim S, Kaldis P (2013). Cdks, cyclins and CKIs: roles beyond cell cycle regulation. *Development*.

[324] Barrera G, Pizzimenti S, Laurora S, Moroni E, Giglioni B, Dianzani MU (2002). 4-hydroxynonenal affects pRb/E2F pathway in HL-60 human leukemic cells. *Biochemical and Biophysical Research Communications*.

[325] Pizzimenti S, Barrera G, Dianzani MU, Brüsselbach S (1999). Inhibition of D1, D2, and A cyclin expression in HL-60 cells by the lipid peroxydation product 4-hydroxynonenal. *Free Radical Biology and Medicine*.

[326] Skorokhod OA, Caione L, Marrocco T (2010). Inhibition of erythropoiesis in malaria anemia: role of hemozoin and hemozoin-generated 4-hydroxynonenal. *Blood*.

[327] Albright CD, Klem E, Shah AA, Gallagher P (2005). Breast cancer cell-targeted oxidative stress: enhancement of cancer cell uptake of conjugated linoleic acid, activation of p53, and inhibition of proliferation. *Experimental and Molecular Pathology*.

[328] Sunjic SB, Cipak A, Rabuzin F, Wildburger R, Zarkovic N (2005). The influence of 4-hydroxy-2-nonenal on proliferation, differentiation and apoptosis of human osteosarcoma cells. *BioFactors*.

[329] Muzio G, Trombetta A, Martinasso G, Canuto RA, Maggiora M (2003). Antisense oligonucleotides against aldehyde dehydrogenase 3 inhibit hepatoma cell proliferation by affecting MAP kinases. *Chemico-Biological Interactions*.

[330] Canuto RA, Muzio G, Ferro M (1999). Inhibition of class-3 aldehyde dehydrogenase and cell growth by restored lipid peroxidation in hepatoma cell lines. *Free Radical Biology and Medicine*.

[331] Pizzimenti S, Barrera G, Calzavara E (2008). Down-regulation of Notch1 expression is involved in HL-60 cell growth inhibition induced by 4-hydroxynonenal, a product of lipid peroxidation. *Medicinal Chemistry*.

[332] Laurora S, Tamagno E, Briatore F (2005). 4-Hydroxynonenal modulation of p53 family gene expression in the SK-N-BE neuroblastoma cell line. *Free Radical Biology and Medicine*.

[333] Barrera G, Martinotti S, Fazio V (1987). Effect of 4-hydroxynonenal on c-myc expression. *Toxicologic Pathology*.

[334] Barrera G, Muraca R, Pizzimenti S (1994). Inhibition of c-myc expression induced by 4-hydroxynonenal, a product of lipid peroxidation, in the HL-60 human leukemic cell line. *Biochemical and Biophysical Research Communications*.

[335] Rinaldi M, Barrera G, Spinsanti P (2001). Growth inhibition and differentiation induction in murine erythroleukemia cells by 4-hydroxynonenal. *Free Radical Research*.

[336] Barrera G, Pizzimenti S, Dianzani MU (2004). 4-Hydroxynonenal and regulation of cell cycle: effects on the pRb/E2F pathway. *Free Radical Biology and Medicine*.

[337] Barrera G, Pizzimenti S, Muraca R (1996). Effect of 4-hydroxynonenal on cell cycle progression and expression of differentiation-associated antigens in HL-60 cells. *Free Radical Biology and Medicine*.

[338] Chaudhary P, Sharma R, Sahu M, Vishwanatha JK, Awasthi S, Awasthi YC (2013). 4-Hydroxynonenal induces G2/M phase cell cycle arrest by activation of the ataxia telangiectasia mutated and Rad3-related protein (ATR)/checkpoint kinase 1 (Chk1) signaling pathway. *Journal of Biological Chemistry*.

[339] Wang X, Yang Y, Moore DR, Nimmo SL, Lightfoot SA, Huycke MM (2012). 4-hydroxy-2-nonenal mediates genotoxicity and bystander effects caused by enterococcus faecalis-infected macrophages. *Gastroenterology*.

[340] Pettazzoni P, Pizzimenti S, Toaldo C (2011). Induction of cell cycle arrest and DNA damage by the HDAC inhibitor panobinostat (LBH589) and the lipid peroxidation end product 4-hydroxynonenal in prostate cancer cells. *Free Radical Biology and Medicine*.

[341] Peng ZF, Koh CHV, Li QT (2007). Deciphering the mechanism of HNE-induced apoptosis in cultured murine cortical neurons: transcriptional responses and cellular pathways. *Neuropharmacology*.

[342] Lee T-J, Lee J-T, Moon S-K, Kim C-H, Park J-W, Kwon TK (2006). Age-related differential growth rate and response to 4-hydroxynonenal in mouse aortic smooth muscle cells. *International Journal of Molecular Medicine*.

[343] Kakishita H, Hattori Y (2001). Vascular smooth muscle cell activation and growth by 4-hydroxynonenal. *Life Sciences*.

[344] Tammali R, Saxena A, Srivastava SK, Ramana KV (2010). Aldose reductase regulates vascular smooth muscle cell proliferation by modulating G1/S phase transition of cell cycle. *Endocrinology*.

[345] Huang C-D, Chen H-H, Wang C-H (2004). Human neutrophil-derived elastase induces airway smooth muscle cell proliferation. *Life Sciences*.

[346] Pizzimenti S, Toaldo C, Pettazzoni P, Dianzani MU, Barrera G (2010). The “two-faced” effects of reactive oxygen species and the lipid peroxidation product 4-hydroxynonenal in the hallmarks of cancer. *Cancers*.

[347] Trachootham D, Alexandre J, Huang P (2009). Targeting cancer cells by ROS-mediated mechanisms: a radical therapeutic approach?. *Nature Reviews Drug Discovery*.

[348] Pelicano H, Carney D, Huang P (2004). ROS stress in cancer cells and therapeutic implications. *Drug Resistance Updates*.

[349] Hileman EO, Liu J, Albitar M, Keating MJ, Huang P (2004). Intrinsic oxidative stress in cancer cells: a biochemical basis for therapeutic selectivity. *Cancer Chemotherapy and Pharmacology*.

[350] Chaudhary P, Sharma R, Sharma A (2010). Mechanisms of 4-hydroxy-2-nonenal induced pro- and anti-apoptotic signaling. *Biochemistry*.

[351] Locksley RM, Killeen N, Lenardo MJ (2001). The TNF and TNF receptor superfamilies: integrating mammalian biology. *Cell*.

[352] Elmore S (2007). Apoptosis: a review of programmed cell death. *Toxicologic Pathology*.

[353] Franklin JL (2011). Redox regulation of the intrinsic pathway in neuronal apoptosis. *Antioxidants and Redox Signaling*.

[354] Haupt S, Berger M, Goldberg Z, Haupt Y (2003). Apoptosis—the p53 network. *Journal of Cell Science*.

[355] Abarikwu SO, Pant AB, Farombi EO (2012). 4-hydroxynonenal induces mitochondrial-mediated apoptosis and oxidative stress in SH-SY5Y human neuronal cells. *Basic and Clinical Pharmacology and Toxicology*.

[356] Sharma A, Sharma R, Chaudhary P (2008). 4-hydroxynonenal induces p53-mediated apoptosis in retinal pigment epithelial cells. *Archives of Biochemistry and Biophysics*.

[357] Sharma R, Sharma A, Dwivedi S, Zimniak P, Awasthi S, Awasthi YC (2008). 4-hydroxynonenal self-limits Fas-mediated DISC-independent apoptosis by promoting export of Daxx from the nucleus to the cytosol and its binding to Fas. *Biochemistry*.

[358] Vaillancourt F, Fahmi H, Shi Q (2008). 4-hydroxynonenal induces apoptosis in human osteoarthritic chondrocytes: the protective role of glutathione-S-transferase. *Arthritis Research and Therapy*.

[359] Awasthi YC, Sharma R, Sharma A (2008). Self-regulatory role of 4-hydroxynonenal in signaling for stress-induced programmed cell death. *Free Radical Biology and Medicine*.

[360] Doorn JA, Petersen DR (2002). Covalent modification of amino acid nucleophiles by the lipid peroxidation products 4-hydroxy-2-nonenal and 4-oxo-2-nonenal. *Chemical Research in Toxicology*.

[361] Sayre LM, Lin D, Yuan Q, Zhu X, Tang X (2006). Protein adducts generated from products of lipid oxidation: focus on HNE and ONE. *Drug Metabolism Reviews*.

[362] Monroy CA, Doorn JA, Roman DL (2013). Modification and functional inhibition of regulator of G-protein signaling 4 (RGS4) by 4-Hydroxy-2-nonenal. *Chemical Research in Toxicology *.

[363] Poli G, Schaur RJ, Siems WG, Leonarduzzi G (2008). 4-hydroxynonenal: a membrane lipid oxidation product of medicinal interest. *Medicinal Research Reviews*.

[364] Choudhury S, Pan J, Amin S, Chung F-L, Roy R (2004). Repair kinetics of trans-4-Hydroxynonenal-induced cyclic 1,N^2^-propanodeoxyguanine DNA adducts by human cell nuclear extracts. *Biochemistry*.

[365] Choudhury S, Dyba M, Pan J, Roy R, Chung FL (2013). Repair kinetics of acrolein- and (E)-4-hydroxy-2-nonenal-derived DNA adducts in human colon cell extracts. *Mutation Research*.

[366] Gros L, Ishchenko AA, Saparbaev M (2003). Enzymology of repair of etheno-adducts. *Mutation Research: Fundamental and Molecular Mechanisms of Mutagenesis*.

[367] Nair J, Srivatanakul P, Haas C (2010). High urinary excretion of lipid peroxidation-derived DNA damage in patients with cancer-prone liver diseases. *Mutation Research: Fundamental and Molecular Mechanisms of Mutagenesis*.

[368] Nair J, Gansauge F, Beger H, Dolara P, Winde G, Bartsch H (2006). Increased etheno-DNA adducts in affected tissues of patients suffering from Crohn’s disease, ulcerative colitis, and chronic pancreatitis. *Antioxidants and Redox Signaling*.

[369] Richard S, Lewis J (2008). *Hazardous Chemicals Desk Reference*.

[370] Ayala A, Parrado J, Bougria M, Machado A (1996). Effect of oxidative stress, produced by cumene hydroperoxide, on the various steps of protein synthesis. Modifications of elongation factor-2. *Journal of Biological Chemistry*.

[371] Parrado J, Bougria M, Ayala A, Castaño A, Machado A (1999). Effects of aging on the various steps of protein synthesis: fragmentation of elongation factor 2. *Free Radical Biology and Medicine*.

[372] Parrado J, Bougria M, Ayala A, MacHado A (1999). Induced mono-(ADP)-ribosylation of rat liver cytosolic proteins by lipid peroxidant agents. *Free Radical Biology and Medicine*.

[373] Parrado J, Absi EH, Machado A, Ayala A (2003). ‘In vitro’ effect of cumene hydroperoxide on hepatic elongation factor-2 and its protection by melatonin. *Biochimica et Biophysica Acta: General Subjects*.

[374] Argüelles S, Machado A, Ayala A (2006). ‘In vitro’ effect of lipid peroxidation metabolites on elongation factor-2. *Biochimica et Biophysica Acta: General Subjects*.

[375] Arguelles S, Cano M, Machado A, Ayala A (2011). Effect of aging and oxidative stress on elongation factor-2 in hypothalamus and hypophysis. *Mechanisms of Ageing and Development*.

[376] Argüelles S, Muñoz MF, Cano M, Machado A, Ayala A (2012). In vitro and in vivo protection by melatonin against the decline of elongation factor-2 caused by lipid peroxidation: preservation of protein synthesis. *Journal of Pineal Research*.

[377] Argüelles S, Machado A, Ayala A (2007). ’In vitro’ protective effect of a hydrophilic vitamin E analogue on the decrease in levels of elongation factor 2 in conditions of oxidative stress. *Gerontology*.

[378] Arguelles S, Cano M, Machado A, Ayala A (2010). Comparative study of the In Vitro protective effects of several antioxidants on elongation factor 2 under oxidative stress conditions. *Bioscience, Biotechnology and Biochemistry*.

[379] Argüelles S, Camandola S, Hutchison ER, Cutler RG, Ayala A, Mattson MP (2013). Molecular control of the amount, subcellular location, and activity state of translation elongation factor 2 in neurons experiencing stress. *Free Radical Biology and Medicine*.

[380] Argüelles S, Camandola S, Cutler RG, Ayala A, Mattson MP (2013). Elongation factor 2 diphthamide is critical for translation of two IRES-dependent protein targets, XIAP and FGF2, under oxidative stress conditions. *Free Radical Biology and Medicine*.

[381] Aboua YG, Brooks N, Mahfouz RZ, Agarwal A, du Plessis SS (2012). A red palm oil diet can reduce the effects of oxidative stress on rat spermatozoa. *Andrologia*.

[382] Kumar TR, Muralidhara M (2007). Induction of oxidative stress by organic hydroperoxides in testis and epididymal sperm of rats in vivo. *Journal of Andrology*.

[383] Chan TS, Shangari N, Wilson JX, Chan H, Butterworth RF, O’Brien PJ (2005). The biosynthesis of ascorbate protects isolated rat hepatocytes from cumene hydroperoxide-mediated oxidative stress. *Free Radical Biology and Medicine*.

[384] Shvedova AA, Kisin ER, Murray AR (2002). Antioxidant balance and free radical generation in vitamin E-deficient mice after dermal exposure to cumene hydroperoxide. *Chemical Research in Toxicology*.

[385] Alam A, Iqbal M, Saleem M, Ahmed S-U, Sultana S (2000). Myrica nagi attenuates cumene hydroperoxide-induced cutaneous oxidative stress and toxicity in Swiss albino mice. *Pharmacology and Toxicology*.

[386] Jamal M, Masood A, Belcastro R (2013). Lipid hydroperoxide formation regulates postnatal rat lung cell apoptosis and alveologenesis. *Free Radical Biology and Medicine*.

[387] Hong CO, Rhee CH, Won NH, Choi HD, Lee KW (2013). Protective effect of 70% ethanolic extract of Lindera obtusiloba Blume on tert-butyl hydroperoxide-induced oxidative hepatotoxicity in rats. *Food and Chemical Toxicology*.

[388] Oh JM, Jung YS, Jeon BS (2012). Evaluation of hepatotoxicity and oxidative stress in rats treated with tert-butyl hydroperoxide. *Food and Chemical Toxicology*.

[389] Kim M-K, Lee H-S, Kim E-J (2007). Protective effect of aqueous extract of Perilla frutescens on tert-butyl hydroperoxide-induced oxidative hepatotoxicity in rats. *Food and Chemical Toxicology*.

[390] Kaur P, Kaur G, Bansal MP (2006). Tertiary-butyl hydroperoxide induced oxidative stress and male reproductive activity in mice: role of transcription factor NF-*κ*B and testicular antioxidant enzymes. *Reproductive Toxicology*.

[391] Liu CL, Wang JM, Chu CY, Cheng MT, Tseng TH (2002). In vivo protective effect of protocatechuic acid on tert-butyl hydroperoxide-induced rat hepatotoxicity. *Food and Chemical Toxicology*.

[392] Hix S, Kadiiska MB, Mason RP, Augusto O (2000). In vivo metabolism of tert-Butyl hydroperoxide to methyl radicals. EPR spin-trapping and DNA methylation studies. *Chemical Research in Toxicology*.

[393] Ma JQ, Ding J, Zhang L, Liu CM (2013). Hepatoprotective properties of sesamin against CCl4 induced oxidative stress-mediated apoptosis in mice via JNK pathway. *Food and Chemical Toxicology*.

[394] Yeh YH, Hsieh YL, Lee YT (2013). Effects of yam peel extract against carbon tetrachloride-induced hepatotoxicity in rats. *Journal of Agricultural and Food Chemistry*.

[395] Knockaert L, Berson A, Ribault C (2012). Carbon tetrachloride-mediated lipid peroxidation induces early mitochondrial alterations in mouse liver. *Laboratory Investigation*.

[396] Choi J-H, Kim D-W, Yun N (2011). Protective effects of hyperoside against carbon tetrachloride-induced liver damage in mice. *Journal of Natural Products*.

[397] Kim H-Y, Kim J-K, Choi J-H (2010). Hepatoprotective effect of pinoresinol on carbon tetrachloride-induced hepatic damage in mice. *Journal of Pharmacological Sciences*.

[398] Wang H, Wei W, Wang N-P (2005). Melatonin ameliorates carbon tetrachloride-induced hepatic fibrogenesis in rats via inhibition of oxidative stress. *Life Sciences*.

[399] Lugo-Huitrón R, Ugalde Muñiz P, Pineda B, Pedraza-Chaverrí J, Ríos C, Pérez-de la Cruz V (2013). Quinolinic acid: an endogenous neurotoxin with multiple targets. *Oxidative Medicine and Cellular Longevity*.

[400] Maldonado PD, Pérez-De La Cruz V, Torres-Ramos M (2012). Selenium-induced antioxidant protection recruits modulation of thioredoxin reductase during excitotoxic/pro-oxidant events in the rat striatum. *Neurochemistry International*.

[401] Sreekala S, Indira M (2009). Impact of co administration of selenium and quinolinic acid in the rat’s brain. *Brain Research*.

[402] Ryu JK, Choi HB, McLarnon JG (2005). Peripheral benzodiazepine receptor ligand PK11195 reduces microglial activation and neuronal death in quinolinic acid-injected rat striatum. *Neurobiology of Disease*.

[403] Rossato JI, Zeni G, Mello CF, Rubin MA, Rocha JBT (2002). Ebselen blocks the quinolinic acid-induced production of thiobarbituric acid reactive species but does not prevent the behavioral alterations produced by intra-striatal quinolinic acid administration in the rat. *Neuroscience Letters*.

[404] Santamaría A, Jiménez-Capdeville ME, Camacho A, Rodríguez-Martínez E, Flores A, Galván-Arzate S (2001). In vivo hydroxyl radical formation after quinolinic acid infusion into rat corpus striatum. *NeuroReport*.

[405] Rodríguez-Martínez E, Camacho A, Maldonado PD (2000). Effect of quinolinic acid on endogenous antioxidants in rat corpus striatum. *Brain Research*.

[406] Jomova K, Valko M (2011). Advances in metal-induced oxidative stress and human disease. *Toxicology*.

[407] Boveris A, Musacco-Sebio R, Ferrarotti N (2012). The acute toxicity of iron and copper: biomolecule oxidation and oxidative damage in rat liver. *Journal of Inorganic Biochemistry*.

[408] Özcelik D, Uzun H, Naziroglu M (2012). N-acetylcysteine attenuates copper overload-induced oxidative injury in brain of rat. *Biological Trace Element Research*.

[409] Alexandrova A, Petrov L, Georgieva A (2008). Effect of copper intoxication on rat liver proteasome activity: relationship with oxidative stress. *Journal of Biochemical and Molecular Toxicology*.

[410] Scharf B, Trombetta LD (2007). The effects of the wood preservative copper dimethyldithiocarbamate in the hippocampus of maternal and newborn Long-Evans rats. *Toxicology Letters*.

[411] Parveen K, Khan MR, Siddiqui WA (2009). Pycnogenol prevents potassium dichromate (K2Cr2O7)-induced oxidative damage and nephrotoxicity in rats. *Chemico-Biological Interactions*.

[412] Kotyzova D, Hodková A, Bludovská M, Eybl V Effect of chromium (VI) exposure on antioxidant defense status and trace element homeostasis in acute experiment in rat.

[413] Karaca S, Eraslan G (2013). The effects of flaxseed oil on cadmium-induced oxidative stress in rats. *Biological Trace Element Research*.

[414] Chen Q, Zhang R, Li W (2013). The protective effect of grape seed procyanidin extract against cadmium-induced renal oxidative damage in mice. *Environmental Toxicology and Pharmacology*.

[415] Leelavinothan P, Kalist S (2011). Beneficial effect of hesperetin on cadmium induced oxidative stress in rats: an in vivo and in vitro study. *European Review for Medical and Pharmacological Sciences*.

[416] Ognjanović BI, Marković SD, Ethordević NZ, Trbojević IS, Stajn AS, Saicić ZS (2010). Cadmium-induced lipid peroxidation and changes in antioxidant defense system in the rat testes: protective role of coenzyme Q(10) and vitamin E. *Reproductive Toxicology*.

[417] Amudha K, Pari L (2011). Beneficial role of naringin, a flavanoid on nickel induced nephrotoxicity in rats. *Chemico-Biological Interactions*.

[418] Pari L, Amudha K (2011). Hepatoprotective role of naringin on nickel-induced toxicity in male Wistar rats. *European Journal of Pharmacology*.

[419] Scibior A, Gołębiowska D, Niedźwiecka I (2013). Magnesium can protect against vanadium-induced lipid peroxidation in the hepatic tissue. *Oxidative Medicine and Cellular Longevity*.

[420] Ścibior A, Zaporowska H, Niedźwiecka I (2010). Lipid peroxidation in the kidney of rats treated with V and/or Mg in drinking water. *Journal of Applied Toxicology*.

[421] Ścibior A, Zaporowska H, Ostrowski J, Banach A (2006). Combined effect of vanadium(V) and chromium(III) on lipid peroxidation in liver and kidney of rats. *Chemico-Biological Interactions*.

[422] Martins EN, Pessano NTC, Leal L (2012). Protective effect of Melissa officinalis aqueous extract against Mn-induced oxidative stress in chronically exposed mice. *Brain Research Bulletin*.

[423] Chtourou Y, Fetoui H, Sefi M (2010). Silymarin, a natural antioxidant, protects cerebral cortex against manganese-induced neurotoxicity in adult rats. *BioMetals*.

[424] Chen MT, Cheng GW, Lin CC, Chen BH, Huang YL (2006). Effects of acute manganese chloride exposure on lipid peroxidation and alteration of trace metals in rat brain. *Biological Trace Element Research*.

[425] Salama SA, Omar HA, Maghrabi IA, Alsaeed MS, El-Tarras AE (2014). Iron supplementation at high altitudes induces inflammation and oxidative injury to lung tissues in rats. *Toxicology and Applied Pharmacology*.

[426] Kim J, Paik HD, Yoon YC, Park E (2013). Whey protein inhibits iron overload-induced oxidative stress in rats. *Journal of Nutritional Science and Vitaminology*.

[427] Arruda LF, Arruda SF, Campos NA, de Valencia FF, de Siqueira EM (2013). Dietary iron concentration may influence aging process by altering oxidative stress in tissues of adult rats. *PloS ONE*.

[428] Yu HC, Feng SF, Chao PL, Lin AMY (2010). Anti-inflammatory effects of pioglitazone on iron-induced oxidative injury in the nigrostriatal dopaminergic system. *Neuropathology and Applied Neurobiology*.

[429] Oktar S, Yönden Z, Aydin M, Ilhan S, Alçin E, Oztürk OH (2009). Protective effects of caffeic acid phenethyl ester on iron-induced liver damage in rats. *Journal of Physiology and Biochemistry*.

[430] Kokoszko A, Dabrowski J, Lewiński A, Karbownik-Lewińska M (2008). Protective effects of GH and IGF-I against iron-induced lipid peroxidation in vivo. *Experimental and Toxicologic Pathology*.

[431] Maharaj DS, Maharaj H, Daya S, Glass BD (2006). Melatonin and 6-hydroxymelatonin protect against iron-induced neurotoxicity. *Journal of Neurochemistry*.

[432] Morales NP, Yamaguchi Y, Murakami K, Kosem N, Utsumi H (2012). Hepatic reduction of carbamoyl-PROXYL in ferric nitrilotriacetate induced iron overloaded mice: an in vivo ESR study. *Biological and Pharmaceutical Bulletin*.

[433] Völkel W, Alvarez-Sánchez R, Weick I, Mally A, Dekant W, Pähler A (2005). Glutathione conjugates of 4-hydroxy-2(E)-nonenal as biomarkers of hepatic oxidative stress-induced lipid peroxidation in rats. *Free Radical Biology and Medicine*.

[434] Eybl V, Kotyzová D, Černá P, Koutenský J (2008). Effect of melatonin, curcumin, quercetin, and resveratrol on acute ferric nitrilotriacetate (Fe-NTA)-induced renal oxidative damage in rat. *Human and Experimental Toxicology*.

